# *N*-(1-Phenylethyl)aziridine-2-carboxylate esters in the synthesis of biologically relevant compounds

**DOI:** 10.3762/bjoc.15.168

**Published:** 2019-07-23

**Authors:** Iwona E Głowacka, Aleksandra Trocha, Andrzej E Wróblewski, Dorota G Piotrowska

**Affiliations:** 1 Bioorganic Chemistry Laboratory, Faculty of Pharmacy, Medical University of Lodz, Muszynskiego 1, 90-151 Lodz, Poland

**Keywords:** alkaloids, amino acids, asymmetric synthesis, ceramides, chiral catalysis, chiral pool, *N*-(1-phenylethyl)aziridine chiron, sphingoids

## Abstract

Since Garner’s aldehyde has several drawbacks, first of all is prone to racemization, alternative three-carbon chirons would be of great value in enantioselective syntheses of natural compounds and/or drugs. This review article summarizes applications of *N*-(1-phenylethyl)aziridine-2-carboxylates, -carbaldehydes and -methanols in syntheses of approved drugs and potential medications as well as of natural products mostly alkaloids but also sphingoids and ceramides and their 1- and 3-deoxy analogues and several hydroxy amino acids and their precursors. Designed strategies provided new procedures to several drugs and alternative approaches to natural products and proved efficiency of a 2-substituted *N*-(1-phenylethyl)aziridine framework as chiron bearing a chiral auxiliary.

## Introduction

The synthesis of enantiomerically pure compounds belongs to the most challenging tasks in organic chemistry for several reasons, just to mention structural studies of natural products or preparation of chiral drugs. They become available by asymmetric synthesis frequently employing chiral synthons (chirons) [1].

**Figure 1:**
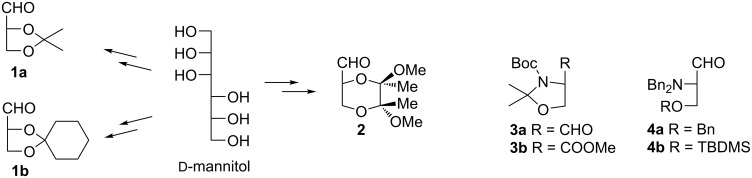
Examples of three-carbon chirons.

Chirons contain functional groups for structural enlargement and at least one stereogenic center which is usually transferred into the final product. To assure the highest possible enantiomeric purity chirons are obtained in most instances from natural products like carbohydrates, amino acids, hydroxy acids or terpenes. The structurally simplest chirons containing three carbon atoms and one stereogenic center can be exemplified by derivatives of ᴅ-glyceraldehyde [2] (2,3-*O*-isopropylidene **1a** [3–4] and 2,3-*O*-cyclohexylidene **1b** [5–6]) and (2*R*,5*R*,6*R*)-5,6-dimethoxy-5,6-dimethyl-1,4-dioxane-2-carbaldehyde (**2**) [7] (Figure 1) which could be prepared from ᴅ-mannitol. While derivatives of glyceraldehyde are configurationally stable Garner’s aldehyde **3a** [8–9], available from ʟ-serine, is configurationally labile and samples with ee as low as 75% can be obtained depending on the reaction conditions. Significant improvement in terms of chemical and configurational stability was achieved by introducing *N*,*N*-dibenzylserinals **4** [10].

**Figure 2:**
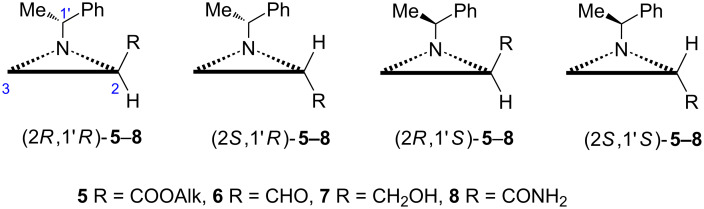
Structures of derivatives of *N*-(1-phenylethyl)aziridine-2-carboxylic acid **5**–**8**.

Another important strategy of asymmetric synthesis relies on chiral auxiliaries, i.e., a specially selected homochiral part of a starting material governing the stereoselectivity of subsequent reactions which is finally easily removed [11]. Among three-carbon chirons related to Garner’s aldehyde derivatives of *N*-(1-phenylethyl)aziridine-2-carboxylic acid **5**–**8** (Figure 2) play an important role in asymmetric synthesis as they function as a chiral synthon combined with a chiral auxiliary [(*R*)- or (*S*)-1-phenylethyl group].

As the closest analogues of Garner’s aldehyde (**3a**) and other chiral α-aminoaldehydes, e.g., **4**, or aziridine aldehydes **6** do not undergo epimerization during preparation as well as in further transformations conducted in the presence of basic reagents because of the high barrier to inversion at the nitrogen in the aziridine ring. The immediate starting materials, esters **5** and methyl ester **3b**, are easier (one step and separation of diastereoisomers for **5** vs three steps for **3b**) to prepare for the aziridine chiron while they are priced comparably. Although DIBAL-H was applied as a reagent of choice in the synthesis of Garner’s aldehyde it has several drawbacks, e.g., overreduction, tricky removal of aluminum salts, a two-step easy to perform sequence (ester **5** to alcohol **7** reduction and re-oxidation to **6**) was generally adapted for aziridine-2-carbaldehydes **6**.

**Figure 3:**
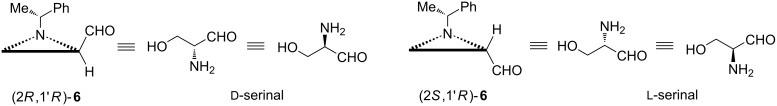
Synthetic equivalency of aziridine aldehydes **6**.

Since the aziridine ring opening with nucleophiles, e.g., oxygen, can relatively easily be achieved [12], and for the structural framework of **5**–**8** occurs with high regioselectivity at the less substituted carbon atom, aldehydes (2*R*,1'*R*)- or (2*R*,1'*S*)-**6** are considered as synthetic equivalents of (*R*)- or ᴅ-serinal while (2*S*,1'*R*)- or (2*S*,1'*S*)-**6** correspond to (*S*)- or ʟ-serinal (Figure 3) [13].

Furthermore, since the aziridine ring openings can be accomplished with other nucleophiles and the reductive cleavage is also known [14–15] the aziridines **5**–**8** offer a plethora of opportunities for enantioselective synthesis of structurally diversified compounds.

In this review we wish to focus attention on applications of **5**–**8** in syntheses of biologically important compounds having a 2-amino-1,3-disubstituted propane unit implanted into their structures because vicinal amino alcohol and 2-aminopropane-1,3-diol scaffolds are present in many natural products as well as compounds of commercial interest as medications.

Synthetic strategies to molecules as simple as amino alcohols to as complex as indolizine alkaloids will be discussed with a special focus on stereoselectivities of key transformations. Whenever possible biological activities of new compounds will be shown to underscore their biological potency. In a few cases mechanistic considerations will be presented to clarify the structural diversity of the products resulted from openings of the aziridine ring. This review was intended to cover the entire literature including patents on (1-phenylethyl)aziridine-2-carboxylic acid and its derivatives till the beginning of 2019. The usefulness of any chiron depends on its availability and to some extent on versatility of the protective groups. In case of the 1-phenylethyl group its removal at any stage of synthesis is not limited to a catalytic hydrogenation but metal-ammonia reduction (Birch reaction) and organic acid in anisole (vide infra) can be efficiently applied. We begin with a short presentation of syntheses of aziridine-2-carboxylates, the corresponding aldehydes and 2-methanols.

## Review

### Syntheses of starting materials

#### Synthesis of *N*-(1-phenylethyl)aziridine-2-carboxylates **5**

**Scheme 1:**
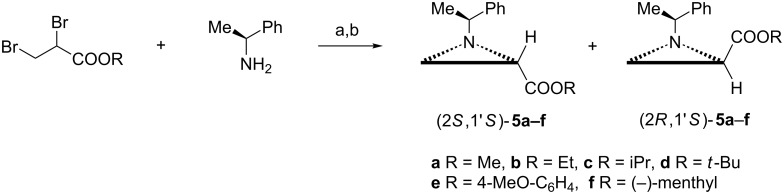
Synthesis of *N*-(1-phenylethyl)aziridine-2-carboxylates **5**. Reagents and conditions: a) TEA, toluene, reflux, 3 h [13]; b) silica gel chromatography or solvent-driven selective crystallization at low-temperature.

In general, enantiomerically pure starting materials used in asymmetric syntheses are prepared from natural products. This does not hold for *N*-(1-phenylethyl)aziridine-2-carboxylates **5** since after synthesis in the Gabriel–Cromwell reaction between 3,3-dibromopropanoate and (*R*)- or (*S*)-1-phenylethylamine (Scheme 1) the separation of mixtures of diastereoisomers (2*R*,1'*R*)- and (2*S*,1'*R*)-**5** as well as (2*R*,1'*S*)- and (2*S*,1'*S*)-**5** has to be accomplished. Fortunately, chromatography appeared a method of choice for esters of aliphatic alcohols: methyl **5a** [16–17], ethyl **5b** [16,18–19], isopropyl **5c** [16] and *tert*-butyl **5d** [16,20] as well as for 4-methoxyphenyl ester **5e** [21].

However, the corresponding (−)-menthyl esters (2*R*,1'*R*)-**5f** and (2*S*,1'*R*)-**5f** were separated by solvent-driven selective crystallization at low-temperature from ethanol and hexane, respectively. The aziridine esters (2*R*,1'*S*)-**5f** and (2*S*,1'*S*)-**5f** were obtained in a similar manner. After transesterification methyl **5a** and ethyl **5b** esters were prepared. Thus, enantiomerically pure *N*-(1-phenylethyl)aziridine-2-carboxylates **5** became commercially available [22].

Other synthetic pathways to esters **5** were elaborated although they are more complex [23–25]. Enzymatically-catalyzed aminolysis of a mixture of (2*R*,1'*R*)-**5a** and (2*S*,1'*R*)-**5a** provided diastereoisomerically pure (de >99%) methyl ester (2*R*,1'*R*)-**5a** and amide (2*S*,1'*R*)-**8** (Figure 2). The same selectivity was observed for (2*R*,1'*S*)-**5a** and (2*S*,1'*S*)-**5a** and both pairs of ethyl esters [26].

When a mixture of *tert*-butyl esters (2*R*,1'*S*)-**5d** and (2*S*,1'*S*)-**5d** was subjected to kinetic resolution in the presence of potassium *tert*-butoxide in tetrahydrofuran (2*R*,1'*S*)-**5d** was produced with low 40% de [27].

**Scheme 2:**
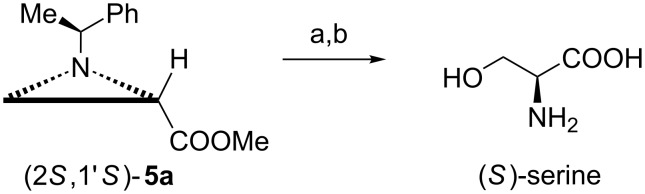
Absolute configuration at C2 in (2*S*,1'*S*)-**5a**. Reagents and conditions: a) 20% HClO_4_, 80 °C, 30 h then Dowex 50 (H^+^); b) H_2_, 20% Pd(OH)_2_/C, EtOH/H_2_O, rt, 24 h.

The absolute configuration at C2 in esters **5** was established by transforming enantiomerically pure (2*R*,1'*S*)-**5a** and (2*S*,1'*S*)-**5a** into (*R*)- and (*S*)-serine, respectively (Scheme 2) [13,16].

#### Synthesis of *N*-(1-phenylethyl)aziridine-2-carbaldehydes **6**

Aziridine aldehydes **6** are in most instances prepared from the corresponding alcohols **7** by Swern oxidation [28–33]. Procedures relying on DIBAL-H reduction of esters **5** are less frequent [34]. Although aziridine aldehydes **6** are chemically stable enough to be chromatographed on silica gel and can later be stored at −10 °C they are normally prepared before use and applied as crude materials.

#### Synthesis of *N*-(1-phenylethyl)aziridine-2-methanols **7**

Aziridine alcohols **7** are usually prepared by LiAlH_4_ reduction of the corresponding esters **5** [22,35–36] although a milder method with a NaBH_4_ and LiCl mixture was also elaborated [37]. A multistep synthesis of (2*S*,1'*R*)-**7** employing the aziridine ring closure as the last step has also been described [38].

### Syntheses of biologically relevant compounds from *N*-(1-phenylethyl)aziridine-2-carboxylate esters

**Scheme 3:**
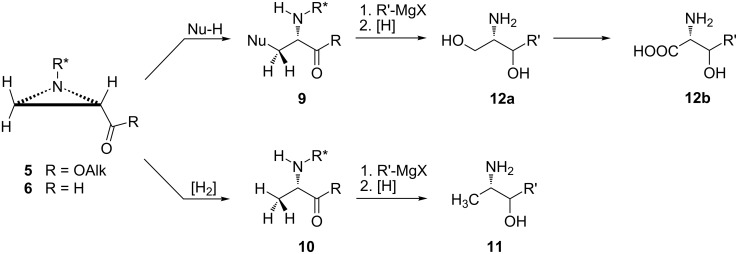
Major synthetic strategies for a 2-ketoaziridine scaffold [R* = (*R*)- or (*S*)-1-phenylethyl; R′ = Alk, Ar].

A 2-ketoaziridine scaffold present in esters **5** and aldehydes **6** can undergo a variety of transformations. Elongation together with further functionalization are possible employing ester and aldehyde groups and the stereochemical outcome of these reactions is controlled by configurations at C2 and at the chiral auxiliary (Scheme 3). The opening of the aziridine ring is expected to occur at the less substituted carbon atom and can be executed with nucleophiles to provide **9** or even by catalytic hydrogenation to form **10**. Thus, biologically important fragments like vicinal amino alcohols **11** or 2-amino-1,3-propanediols **12a** [Nu = OH] can be obtained in highly enantioselective procedures preserving the absolute configuration at C2. The latter compounds are useful precursors to amino acids. Installation of halogen atoms in **9** (Nu = Cl, Br, I) allows for extending of the carbon chain.

**Scheme 4:**
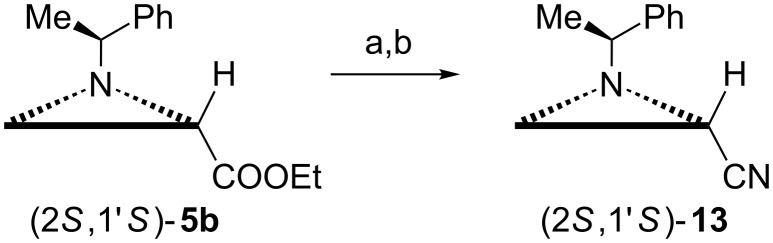
Synthesis of cyanide (2*S*,1'*S*)-**13**. Reagents and conditions: a) NH_3_, EtOH/H_2_O, rt, 72 h; b) Ph_3_P, CCl_4_, TEA, CH_2_Cl_2_, reflux.

Before we start reviewing the synthesis of biologically relevant compounds prepared via opening of the aziridine ring it should be mentioned that cyanides (2*R*,1'*S*)- and (2*S*,1'*S*)-**13** prepared from the respective esters (Scheme 4) **5b** appeared moderately active as immunostimulants [18].

#### Amines and amino alcohols

**By functionalization at C2:** Synthesis of enantiomerically pure amines from 2-substituted *N*-(1-phenylethyl)aziridines **5**–**7** requires a regioselective reductive aziridine ring opening at the less substituted carbon and effective deoxygenation performed at the C2 substituent [39].

**Scheme 5:**
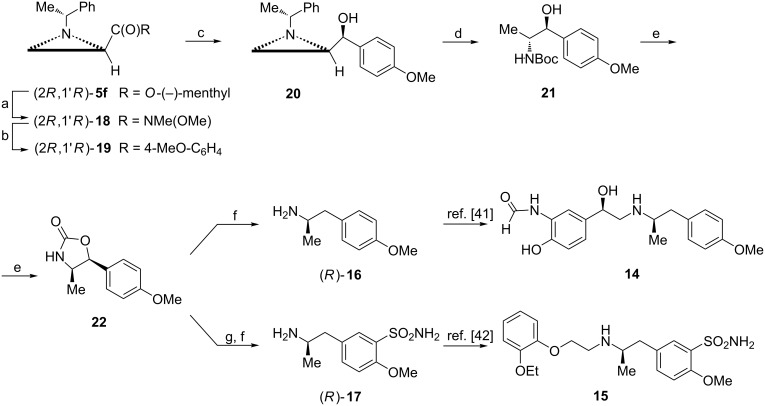
Synthesis of key intermediates (*R*)-**16** and (*R*)-**17** for (*R*,*R*)-formoterol (**14**) and (*R*)-tamsulosin (**15**). Reagents and conditions: a) MeONHMe, iPrMgCl, THF, 0 °C, 0.5 h; b) 4-MeOC_6_H_4_MgBr, THF, −10 °C, 1 h and 0 °C, 0.5 h; c) NaBH_4_, ZnCl_2_, MeOH, −78 °C, 0.5 h; d) H_2_, Pd(OH)_2_, Boc_2_O, EtOH, rt, 8 h; e) NaH, THF, rt, 24 h; f) H_2_, Pd/C, MeOH, rt, 1 h; g) ClSO_3_H, 0 °C, 0.5 h then NH_3_, THF.

This strategy was applied in the formal synthesis of (*R*,*R*)-formoterol (**14**) and (*R*)-tamsulosin (**15**) (Scheme 5) which have been used as therapeutic drugs for many years [40–41]. They are structurally related since they contain an (*R*)-1-aryl-2-propanamine moiety. The synthesis of the respective intermediates (*R*)-**16** and (*R*)-**17** commenced from the ester (2*R*,1'*R*)-**5f** and relied on arylation of Weinreb amide (2*R*,1'*R*)-**18** to afford the aziridine ketone **19**. Its highly stereoselective reduction with the NaBH_4_/ZnCl_2_ mixture (chelation controlled) gave the aziridine alcohol **20** as a major product. Reductive opening of the aziridine ring produced the amino alcohol **21** which was transformed into the substituted oxazolidin-2-one **22**. Its catalytic hydrogenation effected deoxygenation at the benzylic position to supply (*R*)-**16** [42]. To synthesize (*R*)-**17** the aminosulfonyl group was introduced in a standard way before hydrogenation [43].

The synthesis of amino alcohols of general formula **11** (Scheme 3) from 2-substituted *N*-(1-phenylethyl)aziridines **5** and **6** can be achieved by a regioselective reductive aziridine ring opening combined with functionalization of the C2 substituent and optional alkylation or arylation of the nitrogen atom [44].

**Scheme 6:**
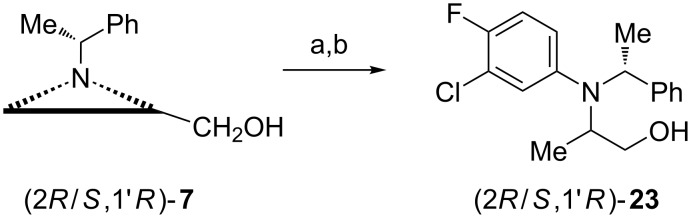
Synthesis of mitotic kinesin inhibitors (2*R*/*S*,1'*R*)-**23**. Reagents and conditions: a) H_2_, Pd(OH)_2_, EtOH, rt; b, 3-chloro-4-fluorophenylboronic acid, Cu(OAc)_2_, pyridine, CH_2_Cl_2_, MS 4 Å, rt, 24 h.

Following a similar regioselective aziridine opening, a mixture of epimeric amino alcohols (2*R*/*S*,1'*R*)-**23** was prepared in two steps from the aziridine alcohol (2*R*/*S*,1'*R*)-**7** (Scheme 6) which was found to be an effective inhibitor of the mitotic kinesin.

**Scheme 7:**
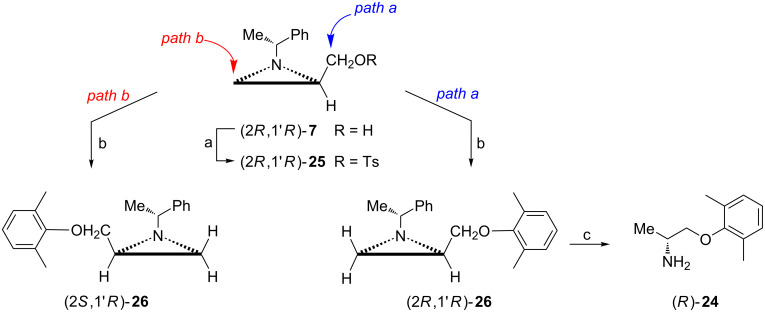
Synthesis of (*R*)-mexiletine ((*R*)-**24**). Reagents and conditions: a) TsCl, TEA, DMAP, CH_2_Cl_2_, rt, 1 h; b) 2,6-dimethylphenol, K_2_CO_3_, DMF/acetone, reflux, 4 h; c) H_2_, Pd/C, MeOH, rt, 12 h.

**Scheme 8:**
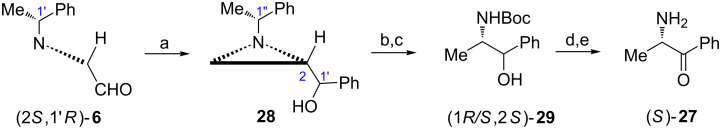
Synthesis of (−)-cathinone ((*S*)-**27**). Reagents and conditions: a) PhMgBr, ether, 0 °C; b) H_2_, 10% Pd(OH)_2_/C, AcOEt, rt, 14 h; c) H_2_, 20% Pd(OH)_2_/C, Boc_2_O, AcOEt, rt, 12 h; d) PCC, CH_2_Cl_2_, rt, 1.5 h; e) 3 N HCl in AcOEt, rt, 0.5 h.

The biologically active enantiomer of mexiletine (*R*)-**24** was efficiently synthesized from the alcohol (2*R*,1'*R*)-**7** (Scheme 7) [45]. When the respective tosylate (2*R*,1'*R*)-**25** was treated with 2,6-dimethylphenoxide two compounds were obtained in a 84:16 ratio. The major ether (2*R*,1'*R*)-**26**, which emerged from the displacement of the tosyloxy group (path a), was accompanied by (2*S*,1'*R*)-**26** which came from the aziridine ring opening at C3 (path b). After chromatographic purification and hydrogenolysis enantiomerically pure (*R*)-**24** was obtained. On the other hand, alkylation of 2,6-dimethylphenol with the tosylate (2*S*,1'*R*)-**25** proceeded regioselectively to give (2*S*,1'*R*)-**26**, a precursor to (*S*)-**24**.

(−)-Cathinone ((*S*)-**27**) is an alkaloid acting as a central nervous system stimulant found in leaves of *Catha edulis*. It was efficiently synthesized from the aldehyde (2*S*,1'*R*)-**6** begining from the addition of phenylmagnesium bromide to give a 4:1 mixture of aziridine alcohols **28** (Scheme 8) [46]. They were subjected a stepwise hydrogenation to form first products of the aziridine ring opening and next *N*-Boc-protected diastereoisomeric amino alcohols (1*R*/*S*,2*S*)-**29** after removal of a chiral auxiliary and finally oxidized to a ketone from which (−)-cathinone ((*S*)-**27**) was isolated as the hydrochloride salt. Starting from the aldehyde (2*R*,1'*S*)-**6** its enantiomer was prepared in a similar way.

Under optimized conditions the diastereoselectivity of the addition of phenyllithium to the aldehyde (2*S*,1'*R*)-**6** exceeded 80% and the major aziridine alcohol (2*S*,1'*S*,1*''R*)-**28** could be separated chromatographically [29]. When (2*S*,1'*S*,1''*R*)-**28** was hydrogenated in the presence of Boc_2_O *N*-protected (−)-norpseudoephedrine (1*S*,2*S*)-(+)-**29** was obtained.

**Scheme 9:**
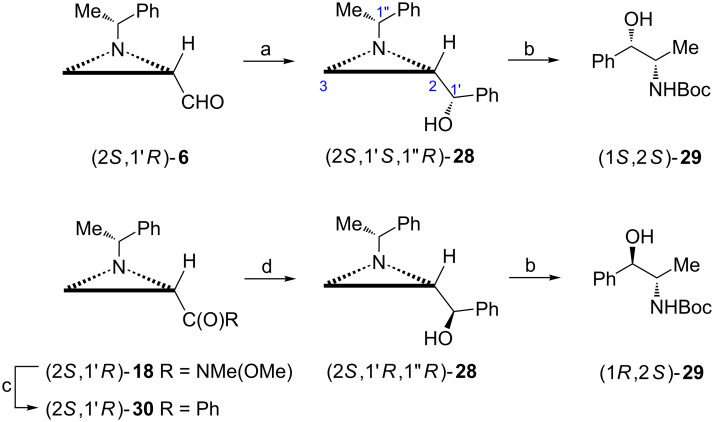
Synthesis of *N*-Boc-norpseudoephedrine ((1*S*,2*S*)-(+)-**29**) and *N*-Boc-norephedrine ((1*R*,2*S*)-**29**). Reagents and conditions: a) PhBr, *t*-BuLi, THF, −78 °C, 2 h; b) H_2_, Pd(OH)_2_/C, AcOEt, rt, 6 h, then Boc_2_O, rt, 6 h; c) PhMgBr, THF, −78 °C, 0.5 h; d) NaBH_4_, ZnCl_2_, MeOH, −78 °C, 0.5 h.

To synthesize *N*-Boc-norephedrine ((1*R*,2*S*)-**29**) the epimeric aziridine alcohol (2*S*,1'*R*,1''*R*)-**28** was needed (Scheme 9) [47]. To this end the aziridine ketone (2*S*,1'*R*)-**30** prepared from Weinreb amide (2*S*,1'*R*)-**18** was reduced with a NaBH_4_/ZnCl_2_ mixture to give almost enantiomerically pure (>99:1) alcohol (2*S*,1'*R*,1''*R*)-**28**. After reductive aziridine ring opening *N*-Boc-norephedrine ((1*R*,2*S*)-**29**) was formed.

**Scheme 10:**
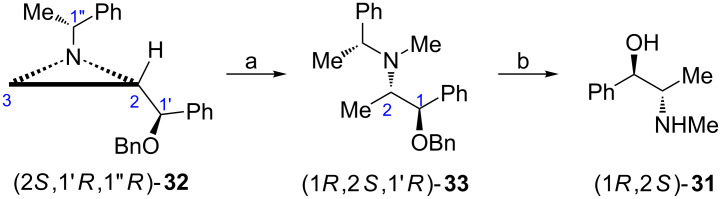
Synthesis of (−)-ephedrine ((1*R*,2*S*)-**31**). Reagents and conditions: a) TfOMe, MeCN then NaBH_3_CN, rt; b) H_2_, Pd(OH)_2_, 50 psi, EtOH, rt.

The synthesis of (−)-ephedrine ((1*R*,2*S*)-**31**) required prior methylation of the nitrogen atom and for this reason it was performed on the benzyl ether (2*S*,1'*R*,1''*R*)-**32** (Scheme 10) [48]. Its reaction with methyl triflate afforded the respective aziridinium ion which was regioselectively reduced to (1*R*,2*S*,1'*R*)-**33**, a protected form of (−)-ephedrine (1*R*,2*S*)-**31**.

**Scheme 11:**
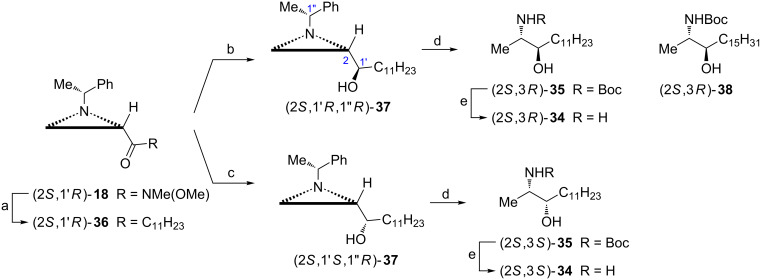
Synthesis of xestoaminol C ((2*S*,3*R*)-**35**), 3-*epi*-xestoaminol C ((2*S*,3*S*)-**35**) and *N*-Boc-spisulosine ((2*S*,3*R*)-**38**). Reagents and conditions: a) C_11_H_23_Br, Mg, THF, 0 °C, 1 h; b) NaBH_4_, ZnCl_2_, MeOH, *−*78 °C, 2 h; c) ʟ-Selectride^®^, THF, −78 °C, 1 h; d) H_2_, Pd(OH)_2_, Boc_2_O, MeOH, rt 6 h; e) HCl, MeOH, reflux, 3 h.

Xestoaminol C ((2*S*,3*R*)-**34**) and 3-*epi*-xestoaminol C ((2*S*,3*S*)-**34**) represent a large family of naturally occurring 1-deoxysphingoids [49]. To finally prove their stereochemistry, they were synthesized from the aziridine ketone (2*S*,1'*R*)-**36** readily available from Weinreb amide (2*S*,1'*R*)-**18** which already contained the required configuration at C2 (Scheme 11) [47]. Introduction of the 3*R* configuration in xestoaminol C and 3*S* in its epimer was achieved by stereoselective reductions with a NaBH_4_/ZnCl_2_ mixture or ʟ-Selectride^®^, respectively, to form aziridine alcohols (2*S*,1'*R*,1''*R*)-**37** and (2*S*,1'*S*,1''*R*)-**37**. Reductive opening of the aziridine ring with concomitant removal of the chiral auxiliary produced protected (2*S*,3*R*)-**35** and (2*S*,3*S*)-**35** which were hydrolyzed to xestoaminol C ((2*S*,3*R*)-**34**) and 3-*epi*-xestoaminol C ((2*S*,3*S*)-**34**). Homologous *N*-Boc-spisulosine ((2*S*,3*R*)-**38**) was acquired following a similar procedure [47].

**Scheme 12:**
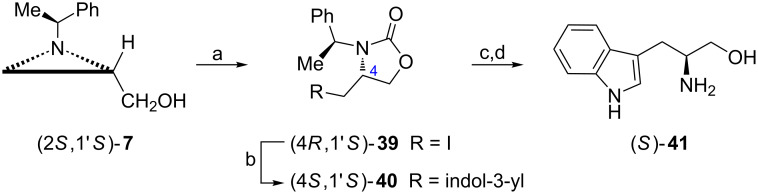
Synthesis of ʟ-tryptophanol ((*S*)-**41**). Reagents and conditions: a) CDI, MeCN, rt, 1 h then TMSI, MeCN, rt, 3 h; b) indole, EtMgBr, toluene/THF, reflux, 3 h; c) Li, NH_3_, THF, −78 °C, 0.5 h; d) LiOH, EtOH/H_2_O, reflux, 2 h.

**By functionalization at C3:** In the presence of carbonyldiimidazole (CDI) and iodotrimethylsilane the aziridine alcohol (2*S*,1'*S*)-**7** was transformed into the 4-(iodomethyl)oxazolidin-2-one (4*R*,1'*S*)-**39** via a regioselective opening with iodide and cyclization (Scheme 12) [50]. As expected alkylation of the indole ring with (4*R*,1'*S*)-**39** occurred at C3 to give (4*S*,1'*S*)-**40** which was deprotected in two steps (Birch reduction and alkaline hydrolysis) to provide ʟ-tryptophanol ((*S*)-**41**).

**Scheme 13:**
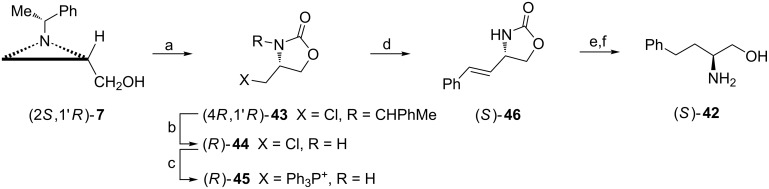
Synthesis of ʟ-homophenylalaninol ((*S*)-**42**). Reagents and conditions: a) NaH, THF, 0 °C to −78 °C, 1 h then phosgene/toluene, −78 °C, 2 h; b) anisole, MeSO_3_H, hexane, reflux, 4 h; c) Ph_3_P, NaI, DMF, 100 °C, 24 h; d) LiHMDS, THF, −78 °C then PhCHO, −78 °C to rt, 2 h; e) H_2_, 5% Pd/C, EtOH, rt, 10 h; f) LiOH, EtOH, reflux, 2 h.

The successful transformation of the aziridine alcohol (2*S*,1'*R*)-**7** into ʟ-homophenylalaninol ((*S*)-**42**) depended on two crucial steps: the aziridine ring opening in the presence of phosgene to form the 4-(chloromethyl)oxazolidin-2-one (4*R*,1'*R*)-**43** and Wittig olefination to add the benzyl moiety (Scheme 13) [51]. Before conversion of the chloromethyl group into a phosphonium salt the chiral auxiliary was removed. The reaction of the ylide (*R*)-**45** prepared from the chloride (*R*)-**44** with benzaldehyde afforded (*E*)-alkene (*S*)-**46** which after hydrogenation and basic hydrolysis gave ʟ-homophenylalaninol ((*S*)-**42**).

**Scheme 14:**
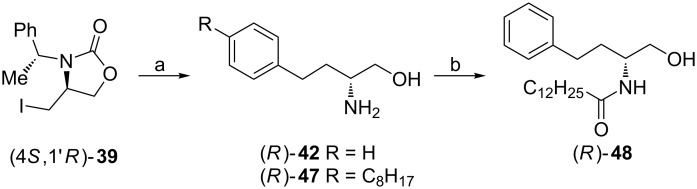
Synthesis of ᴅ-homo(4-octylphenyl)alaninol ((*R*)-**47**) and a sphingolipid analogue (*R*)-**48**. Reagents and conditions: a) see Scheme 13, steps b–f; b) C_12_H_25_COCl, NaOH, H_2_O, rt.

ᴅ-Homophenylalaninol ((*R*)-**42**) and its homolog containing a 4-octylphenyl group (*R*)-**47** are of interest in the synthesis of 3-deoxysphingoid analogues [52–53]. For this reason their syntheses began from the 4-(iodomethyl)oxazolidin-2-one (4*S*,1'*R*)-**39** prepared from the aziridine alcohol (2*R*,1'*R*)-**7** as shown on Scheme 12 and involved formation of the amino alcohols (*R*)-**42** and (*R*)-**47** employing procedures described in Scheme 14. Both (*R*)-**47** and (*R*)-**48** appeared moderate inhibitors of human sphingosine kinases hSphK1 and hSphK2 at 50 μM concentrations as compared to *N*,*N*-dimethylsphingosine (DMS) [52].

**Scheme 15:**
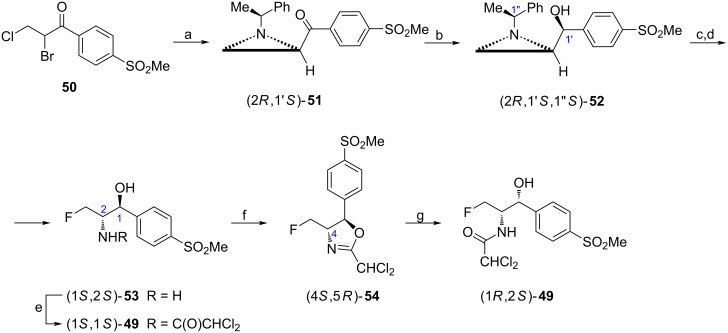
Synthesis of florfenicol ((1*R*,2*S*)-**49**). Reagents and conditions: a) (*S*)-1-phenylethylamine, TEA, MeOH, rt, 3 h then crystallization; b) NaBH_4_, ZnCl_2_, MeOH, −40 °C, 4 h; c) Et_3_N·3HF, ClCH_2_CH_2_Cl, reflux, 3 h; d) H_2_, 10% Pd/C, MeOH, 1.2 atm, 40 °C; e) Cl_2_CHCOOMe, TEA, MeOH, 50 °C, 5 h; f) MsCl, TEA, CH_2_Cl_2_, rt, overnight; g) H_2_O, iPrOH, 80 °C, 1 h.

**By functionalization at C2 and C3:** Florfenicol ((1*R*,2*S*)-**49**) is used as antimicrobial in veterinary medicine [54]. Recently another strategy which applied the (1-phenylethyl)aziridine chemistry has been disclosed (Scheme 15) [55]. The aziridine ketone (2*R*,1'*S*)-**51** (de 98.8%) was obtained when ketone **50** was reacted with (*S*)-1-phenylethylamine. Its reduction with a NaBH_4_/ZnCl_2_ mixture gave the alcohol (2*R*,1'*S*,1''*S*)-**52** (de 92%) which was subjected to the aziridine ring opening with HF followed by the removal of the chiral auxiliary to afford (1*S*,2*S*)-**53** (de 99.1%). Dichloroacetylation led to the formation of (1*S*,2*S*)-**49** and provided a basis for the inversion of configuration at C2 by *O*-mesylation and intramolecular displacement to yield the 2-oxazoline (4*S*,5*R*)-**54** readily hydrolyzed to florfenicol ((1*R*,2*S*)-**49**).

**Scheme 16:**
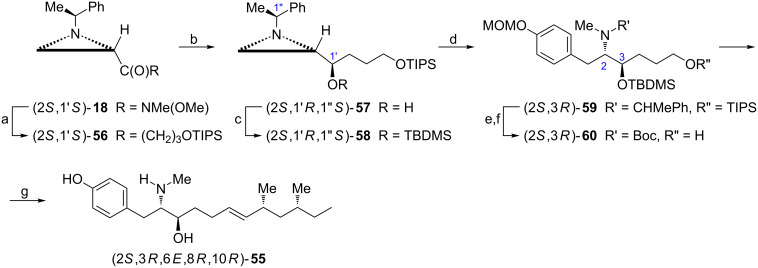
Synthesis of natural tyroscherin ((2*S*,3*R*,6*E*,8*R*,10*R*)-**55**). Reagents and conditions: a) I(CH_2_)_3_OTIPS, *t*-BuLi, ether, −78 °C to rt, 2 h; b) NaBH_4_, ZnCl_2_, MeOH, −78 °C, 0.5 h; c) TBDMSCl, DMAP, CH_2_Cl_2_, rt, 12 h; d) TfOMe, CuI, dioxane, rt, 0.5 h then 4-MOMOC_6_H_4_MgBr, THF, 0 °C, 10 min.; e) Et_3_N·HF, pyridine, rt, 26 h; f) H_2_, Pd(OH)_2_, EtOH, rt, 3 h then Boc_2_O, rt, 1 h; g) see ref. [56].

Naturally occurring tyroscherin ((2*S*,3*R*,6*E*,8*R*,10*R*)-**55**) was recognized for its anticancer activity [56]. Alkylation of Weinreb amide (2*S*,1'*S*)-**18** and stereoselective reduction of the corresponding ketone (2*S*,1'*S*)-**56** with the NaBH_4_/ZnCl_2_ mixture gave the aziridine alcohol (2*S*,1'*R*,1''*S*)-**57** already containing exact absolute configurations (2*S* and 3*R*) of the final product (Scheme 16) [57]. A two-step aziridine ring opening was performed on the silylated alcohol (2*S*,1'*R*,1''*S*)-**58** and included *N*-methylation and reaction with the protected phenylmagnesium bromide to form (2*S*,3*R*)-**59**. The last step opened the way to synthesis of tyroscherin analogues [58]. The alcohol (2*S*,3*R*)-**60** was obtained after selective desilylation and *N*-debenzylation with simultaneous *N*-Boc protection and served as a key intermediate for installation of a dimethylhexylene fragment in Julia–Kocienski olefination [56].

**Scheme 17:**
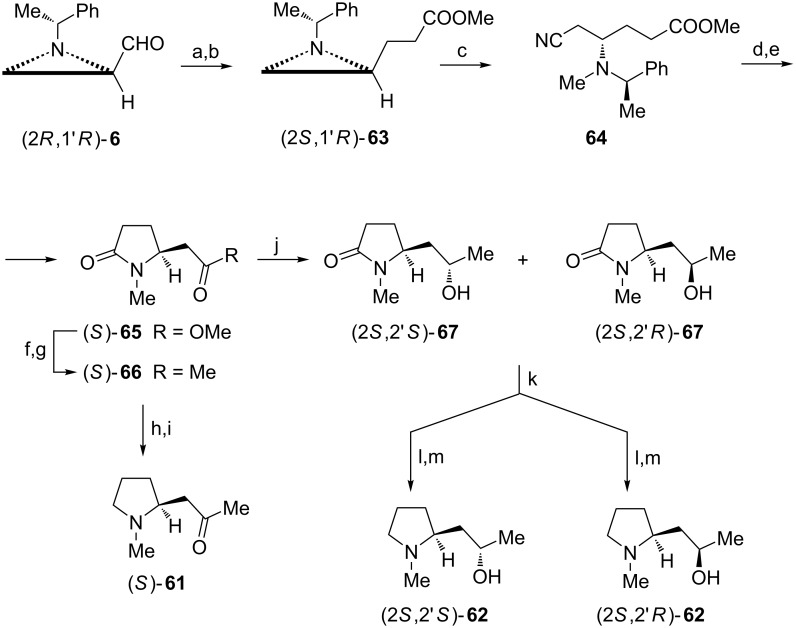
Syntheses of (−)-hygrine (*S*)-**61**, (−)-hygroline (2*S*,2'*S*)-**62** and (−)-pseudohygroline (2*S*,2'*R*)-**62**. Reagents and conditions: a) Ph_3_P=CHCOOMe, MeOH, 0 °C, 1 h; b) 2-nitrobenzenesulfonyl hydrazide (NBSH), TEA, CH_2_Cl_2_, 0 °C to rt, 12 h; c) TfOMe, NaCN, MeCN, 0 °C to rt, 1 h; d) HCl, reflux, 1 h then MeOH, H_2_SO_4_, reflux, 8 h; e) H_2_, Pd(OH)_2_, MeOH, rt, 6 h; f) MeONHMe, Me_3_Al, CH_2_Cl_2_, 0 °C, 1 h; g) MeMgBr, THF, −78 °C to −45 °C, 4 h; h) HC(OMe)_3_, PTSA, MeOH, 40 °C, 1.5 h; i) LiAlH_4_, THF, reflux, 1 h then 6 M HCl; j) NaBH_4_, CoCl_2_, MeOH, −78 °C, 1.5 h and rt, 1 h; k) TBDPSCl, imidazole, DMAP, CH_2_Cl_2_, rt, 1 h; l) LiAlH_4_, THF, reflux, 1 h; m) see ref. [63].

Pyrrolidine alkaloids (−)-hygrine ((*S*)-**61**) and (−)-hygroline ((2*S*,2'*S*)-**62**) were isolated from many natural sources and are known as precursors to tropane alkaloids [59]. Together with (−)-pseudohygroline (2*S*,2'*R*)-**62** they were synthesized from a common intermediate (2*S*,1'*R*)-**63** prepared from the aldehyde (2*R*,1'*R*)-**6** by Wittig olefination [60] and a regioselective C=C bond reduction (Scheme 17) [61]. *N*-Methylation was followed by the aziridinium ion opening with cyanide to access compound **64** which was first transformed into diester and later after hydrogenolysis into the γ-lactam (*S*)-**65** [62]. The methoxycarbonyl to acetyl group conversion was accomplished via Weinreb amide to give the pyrrolidin-2-one (*S*)-**66**. Acetal formation, reduction of the amide function and deprotection completed synthesis of (−)-hygrine (*S*)-**61**. To synthesize (−)-hygroline (2*S*,2'*S*)-**62** and (−)-pseudohygroline (2*S*,2'*R*)-**62** the carbonyl group in (*S*)-**66** was reduced and the diastereoisomeric alcohols (2*S*,2'*S*)-**67** and (2*S*,2'*R*)-**67** were separated as *tert*-butyldiphenylsilyl ethers, individually transformed into 2-(3-hydroxypropyl)pyrrolidines by LiAlH_4_ reduction and deprotected.

#### Diamines

**Scheme 18:**
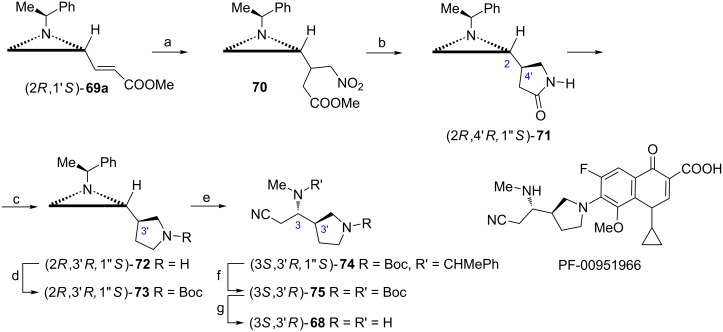
Synthesis of pyrrolidine (3*S*,3'*R*)-**68**, a fragment of the fluoroquinolone antibiotic PF-00951966. Reagents and conditions: a) MeNO_2_, TBAF, THF, rt, 4 h; b) H_2_, 10% PtO_2_, MeOH, rt, 48 h; c) LiAlH_4_, THF, reflux, 1 h; d) Boc_2_O, EtOH, rt, 6 h; e) TfOMe, NaCN, MeCN, 0 °C to rt; f) H_2_, 10% Pd/C, Boc_2_O, THF, rt; g) CF_3_COOH, CH_2_Cl_2_, rt, 2 h.

PF-00951966 (Scheme 18) belongs to a fluoroquinolone family of antibiotics which is substituted at C7 with a (3*R*)-3-[(1*S*)-2-cyano-1-(methylamino)ethyl]pyrrolidin-1-yl group [64]. The synthesis of the corresponding pyrrolidine (3*S*,3'*R*)-**68** began from the aziridine (*E*)-acrylate (2*R*,1'*S*)-**69a** (Scheme 18) readily prepared from the aldehyde (2*S*,1'*S*)-**6** [31]. Michael addition of nitromethane to the acrylate (2*R*,1'*S*)-**69a** gave an inseparable mixture (70:30) of aziridine esters **70** which were subjected to a selective reduction of the nitro group to afford the major pyrrolidine-2-one (2*R*,4'*R*,1''*S*)-**71** after chromatographic purification [65]. The reduction of the amide bond gave (2*R*,3'*R*,1''*S*)-**72** thus providing a cyclic part of (3*S*,3'*R*)-**68**. The transformation of the aziridine moiety into the 2-cyano-1-(methylamino)ethyl group was carried out after *N*-Boc protection and involved methylation of (2*R*,3'*R*,1''*S*)-**73** with simultaneous opening of the aziridinium ion with cyanide to form (3*S*,3'*R*,1''*S*)-**74** with high (97:3) regioselectivity. The removal of the chiral auxiliary afforded a stable di-*N*-Boc derivative (3*S*,3'*R*)-**75** which was transformed into (3*S*,3'*R*)-**68** in the presence of acids.

**Scheme 19:**
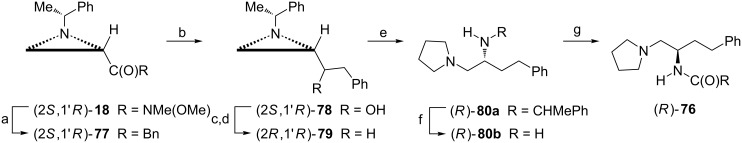
Synthesis of sphingolipid analogues (*R*)-**76**. Reagents and conditions: a) BnBr, Mg, THF, reflux, 6 h; b) LiAlH_4_, THF, 0 °C, 1 h; c) MsCl, TEA, CH_2_Cl_2_, 0 °C, 1 h; d) LiAlH_4_; e) TMSCl, NaI, MeCN, rt, 2 h then pyrrolidine, reflux, 2 h; f) H_2_, Pd(OH)_2_, AcOH, EtOH, rt; g) RC(O)Cl, NaOH, THF/H_2_O, rt.

In search for a sphingolipid analogue synthesis of compounds of general formula (*R*)-**76** was undertaken (Scheme 19) [53]. The aziridine ketone (2*S*,1'*R*)-**77** obtained from Weinreb amide (2*S*,1'*R*)-**18** was subjected to a three-step deoxygenation (reduction, mesylation of the alcohol (2*S*,1'*R*)-**78** and reduction of the mesylate) to locate the 2-phenylethyl substituent at C2. Installation of the pyrrolidine ring at C3 was achieved after opening of the aziridine ring with iodotrimethylsilane to give a protected diamine (*R*)-**80a**. Removal of the chiral auxiliary yielded the diamine (*R*)-**80b** which after acylation provided analogues (*R*)-**76**. Compound (*R*)-**76** (R = C_9_H_19_) appeared inactive as inhibitor of human sphingosine kinases 1 and 2.

#### 1,2-Diamino-3-hydroxy derivatives

**Scheme 20:**
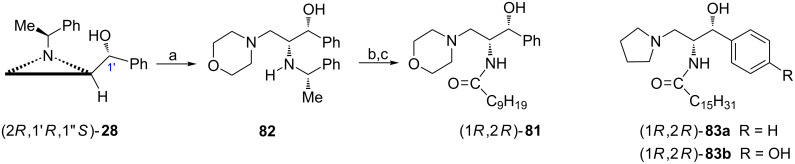
Synthesis of ᴅ-*threo*-PDMP (1*R*,2*R*)-**81**. Reagents and conditions: a) TMSCl, NaI, MeCN, rt, 1 h 50 min, then morpholine, reflux, 2 h; b) H_2_, 10% Pd(OH)_2_, AcOH, 40 °C, 4 h; c) Me(CH_2_)_8_COCl, NaOH, THF/H_2_O, rt.

The interest in sphingoid analogues stems from the involvement of sphingolipid metabolites in an array of important cell processes. ᴅ-*threo*-PDMP (1*R*,2*R*)-**81** is a ceramide analogue identified as an inhibitor of glucosylceramide synthase (GCS) at micromolar concentrations [66–67]. It was efficiently synthesized [68–69] employing the alcohol (2*R*,1′*R*,1''*S*)-**28** prepared from the aziridine aldehyde (2*R*,1′*S*)-**6** which provided the 2*R* absolute configuration of the final product while the configuration at C1' was created by a stereoselective addition of phenylmagnesium bromide (Scheme 20) [29]. The aziridine ring opening in (2*R*,1′*R*,1''*S*)-**28** with morpholine first required the reaction with iodotrimethylsilane and furnished diamino alcohol **82** which after *N*-debenzylation and acylation gave (1*R*,2*R*)-**81**.

In a similar way other GCS inhibitors, e.g., ᴅ-*threo*-P4 (1*R*,2*R*)-**83a** and ᴅ-*threo*-4'-hydroxy-P4 (1*R*,2*R*)-**83b** (Scheme 20) were obtained and examined as potential medications for treating cognitive disorders [70], chronic pain [71] or as immunostimulants [72].

**Scheme 21:**
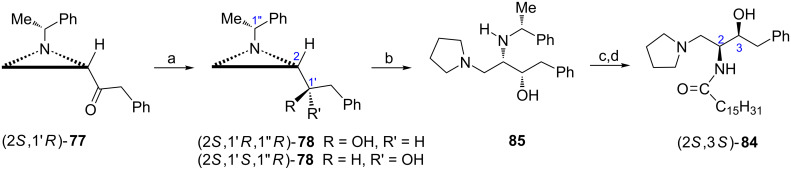
Synthesis of the sphingolipid analogue SG-14 (2*S*,3*S*)-**84**. Reagents and conditions: a) LiAlH_4_, THF, 0 °C, 1 h then separation of diastereoisomers; b) TMSCl, NaI, MeCN, rt, 2 h then pyrrolidine, reflux, 2 h; c) H_2_, Pd(OH)_2_, AcOH, EtOH, rt; d) Me(CH_2_)_14_COCl, NaOH, THF/H_2_O, rt.

Homologues of ʟ-*threo*-P4, e.g., compound (2*S*,3*S*)-**84** were synthesized following the strategy depicted on a previous scheme [52–53]. Reduction of the aziridine ketone (2*S*,1′*R*)-**77** with LiAlH_4_ afforded a mixture of diastereoisomeric alcohols (2*S*,1'*R*,1''*R*)-**78** and (2*S*,1'*S*,1''*R*)-**78** with low (up to 33%) diastereoselectivity readily separable chromatographically. The sphingolipid analogue SG-14 (2*S*,3*S*)-**84** was obtained from (2*S*,1'*S*,1''*R*)-**78** as already described (Scheme 21) and was found to inhibit hSphK2 with the same potency as *N*,*N*-dimethylsphingosine (DMS) being completely inactive toward hSphK1.

#### 2-Amino-1,3-diols

**Scheme 22:**
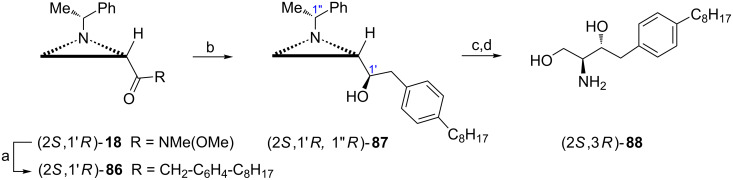
Synthesis of the sphingolipid analogue SG-12 (2*S*,3*R*)-**88**. Reagents and conditions: a) 1-(bromomethyl)-4-octylbenzene, Mg, THF, 50 °C, 6 h; b) LiAlH_4_, THF, 0 °C, 1 h then separation of diastereoisomers; c) AcOH, CH_2_Cl_2_, rt, 18 h then KOH, MeOH, rt, 2 h; d) H_2_, Pd(OH)_2_, EtOH, 100 psi, rt.

A 2-amino-1,3-dihydroxypropyl fragment **12** of sphingosine and ceramides of the required 2*S*,3*R* configuration can also originate from the aziridine alcohol, e.g., (2*S*,1'*R*,1''*R*)-**87** prepared from the ketone (2*S*,1'*R*)-**86** via Weinreb amide (2*S*,1'*R*)-**18** [52–53]. The aziridine ring opening in (2*S*,1'*R*,1''*R*)-**87** with acetic acid followed by hydrolysis and hydrogenolytic removal of the chiral auxiliary gave the sphingosine analogue SG-12 (2*S*,3*R*)-**88** (Scheme 22) as selective and potent as the ceramide analogue SG-14 (2*S*,3*S*)-**84** in inhibiting hSphK2.

**Scheme 23:**
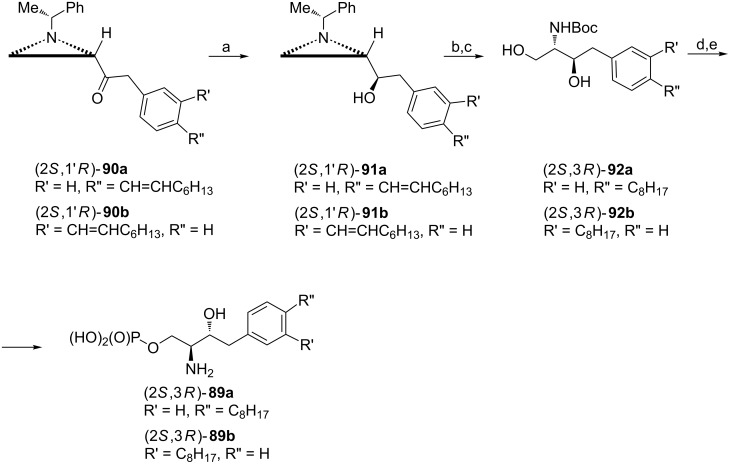
Synthesis of sphingosine-1-phosphate analogues DS-SG-44 and DS-SG-45 (2*S*,3*R*)-**89a** and (2*S*,3*R*)-**89a**. Reagents and conditions: a) NaBH_4_, ZnCl_2_, MeOH, −78 °C; b) AcOH, CH_2_Cl_2_, rt then KOH, EtOH, rt; c) H_2_, Pd(OH)_2_, 100 psi, Boc_2_O, rt; d) P(OMe)_3_, CBr_4_, pyridine, 0 °C; e) TMSBr, CH_2_Cl_2_, rt then H_2_O, rt.

Sphingosine-1-phosphate (S1P) analogues (2*S*,3*R*)-**89a** and (2*S*,3*R*)-**89b** with 1,4- and 1,3-disubstituted benzene rings incorporated into the alkyl chain were obtained from aziridine ketones (2*S*,1'*R*)-**90a** and (2*S*,1'*R*)-**90b** (Scheme 23) readily prepared using Weinreb amide (2*S*,1'*R*)-**18** which introduced the correct 2*S* configuration into the final product [73]. The highly stereoselective reduction of ketones **90a** and **90b** with a NaBH_4_/ZnCl_2_ mixture gave aziridine alcohols **91a** and **91b** having the required 3*R* configuration of the final product. The terminal hydroxymethyl group was acquired as shown earlier to provide (2*S*,3*R*)-**92a** and (2*S*,3*R*)-**92b** as *N*-Boc derivatives after *N*-debenzylation. Analogues (2*S*,3*R*)-**89a** (DS-SG-44) and (2*S*,3*R*)-**89b** (DS-SG-45) were formed after a regioselective phosphorylation and final treatment with bromotrimethylsilane. DS-SG-44 emerged as an agonist of S1P receptors while DS-SG-45 was found inactive.

**Scheme 24:**
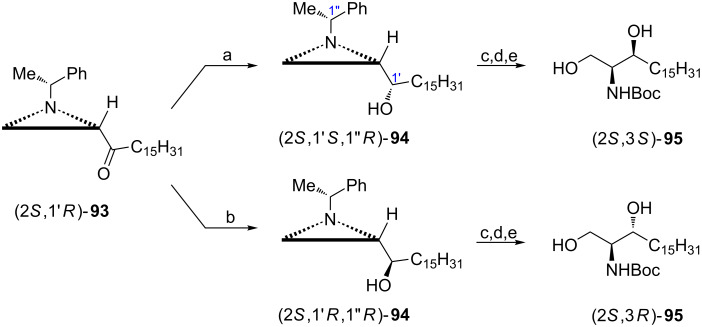
Synthesis of *N*-Boc-safingol ((2*S*,3*S*)-**95**) and *N*-Boc-ᴅ-*erythro*-sphinganine ((2*S*,3*R*)-**95**). Reagents and conditions: a) ʟ-Selectride^®^, THF, −70 °C, 0.5 h; b) NaBH_4_, ZnCl_2_, MeOH, −30 °C, 0.5 h; c) AcOH, CH_2_Cl_2_, rt, 14 h; d) KOH, EtOH, rt, 3 h; e) H_2_, Pd(OH)_2_/C, Boc_2_O, MeOH, rt, 15 h.

Dihydrosphingosines, e.g., safingol and sphinganine itself or as components of dihydroceramides are of interest as enzyme inhibitors [74–75]. Their common vicinal aminohydroxy fragment was efficiently synthesized from the aziridine ketone (2*S*,1'*R*)-**93** readily prepared from the ester (2*S*,1'*R*)-**5f** [47]. To secure the 3*S* configuration in *N*-Boc-safingol the ketone (2*S*,1'*R*)-**93** was reduced with ʟ-Selectride^®^ giving the (*S*)-alcohol (2*S*,1'*S*,1''*R*)-**94** stereoselectively (Scheme 24). On the other hand, the 3*R* configuration in ᴅ-*erythro*-sphinganine was assured when a NaBH_4_/ZnCl_2_ mixture was applied in a chelation-controlled reduction of the ketone (2*S*,1'*R*)-**93** to provide the (*R*)-alcohol (2*S*,1'*R*,1''*R*)-**94** as a major (94:6) product. Alcohols (2*S*,1'*S*,1''*R*)- and (2*S*,1'*R*,1''*R*)-**94** were transformed into *N*-Boc-safingol (2*S*,3*S*)-**95** and *N*-Boc-ᴅ-*erythro*-sphinganine (2*S*,3*R*)-**95** in three standard steps.

**Scheme 25:**
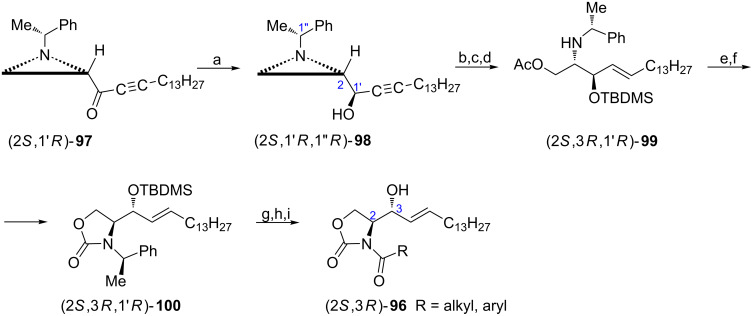
Synthesis of ceramide analogues (2*S*,3*R*)-**96**. Reagents and conditions: a) NaBH_4_, ZnCl_2_, MeOH, −78 °C, 0.5 h; b) LiAlH_4_, THF, rt, 3 h; c) TBDMSCl, DMAP, CH_2_Cl_2_, rt, 17 h; d) AcOH, CH_2_Cl_2_, rt, 10 h; e) KOH, EtOH, rt, 1 h; f) CDI, DBU, CH_2_Cl_2_, rt, overnight; g) Li, NH_3_ liquid, *t*-BuOH, THF, −78 °C, 0.5 h; h) RCOCl, NaHMDS, THF, −78 °C; i) TBAF, THF, 0 °C, 4 h.

Ceramide and other sphingolipids are mainly localized in cell membranes and are involved in important cell processes. A series of constrained analogues of ceramide (2*S*,3*R*)-**96** modified by converting the terminal aminohydroxy fragment into oxazolidin-2-one and acylation of the nitrogen atom with 13 selected acyl chlorides was synthesized starting from the aziridine ketone (2*S*,1'*R*)-**97** (Scheme 25) [76]. To introduce the *R* absolute configuration at C3 (ceramide numbering) the aziridine alcohol (2*S*,1'*R*,1''*R*)-**98** was stereoselectively obtained by reduction of the corresponding ketone with a NaBH_4_/ZnCl_2_ mixture. Treatment of (2*S*,1'*R*,1''*R*)-**98** with LiAlH_4_ to secure the *trans*-configured alkene, protection of the hydroxy group and the regioselective aziridine ring opening led to the formation of the acetate (2*S*,3*R*,1'*R*)-**99**. After basic hydrolysis the oxazolidin-2-one (2*S*,3*R*,1'*R*)-**100** was produced which was later transformed into the ceramide analogues (2*S*,3*R*)-**96** in three standard steps. From a series of 13 modified ceramides the analogue containing the *N*-cyclopentylcarbonyl group appeared more active against human leukemia HL-60 cells than natural *N*-acetylceramide. This observation prompted to synthesize a next series of analogues retaining the *N*-cyclopentycarbonyl moiety while modifying the alkyl chain. It appeared that an analogue having a shorter chain (C_10_H_21_ instead of C_13_H_27_) was slightly less active than (2*S*,3*R*)-**96** (R = cyclopentyl) but still more active than *N*-acetylceramide.

**Scheme 26:**
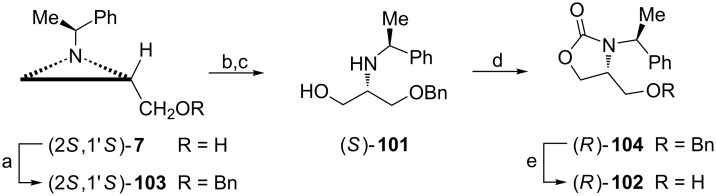
Synthesis of orthogonally protected serinols, (*S*)-**101** and (*R*)-**102**. Reagents and conditions: a) BnBr, NaH, TBAI, THF, rt, 22 h; b) AcOH, CHCl_3_, reflux, 6 h; c) KOH, EtOH, reflux, 0.5 h; d) CDI, CHCl_3_, 50 °C, 17 h; e) H_2_, Pd(OH)_2_, AcOEt/MeOH, rt, 2 h.

A 2-amino-1,3-dihydroxypropyl fragment **12** can also be found in serinol and 3-phenylserinol, potential precursors to the respective amino acids. Orthogonally protected serinols (*S*)-**101** and (*R*)-**102** were synthesized from the benzyl ether **103** utilizing the aziridine alcohol (2*S*,1'*S*)-**7** (Scheme 26) [77]. The aziridine ring opening with acetic acid and saponification led to the formation of (*S*)-**101**. Protection of the vicinal aminohydroxy fragment as an oxazolidin-2-one provided (*R*)-**104** which after selective debenzylation produced (*R*)-**102**.

**Scheme 27:**
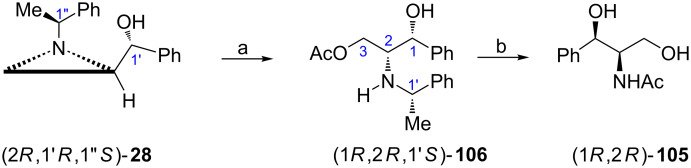
Synthesis of *N*-acetyl-3-phenylserinol ((1*R*,2*R*)-**105**). Reagents and conditions: a) AcOH, CH_2_Cl_2_, reflux, 4 h; b) H_2_, Pd(OH)_2_, AcOEt/MeOH, rt, 48 h.

The synthesis of *N*-acetyl-3-phenylserinol ((1*R*,2*R*)-**105**) was readily accomplished [78] from the aziridine alcohol (2*R*,1'*R*,1''*S*)-**28** available by addition of phenyllithium to the aldehyde (2*R*,1'*S*)-**6** [30]. Opening of the aziridine ring in (2*R*,1'*R*,1''*S*)-**28** with acetic acid yielded the acetate (1*R*,2*R*,1'*S*)-**106** while catalytic hydrogenolysis led to the formation of (1*R*,2*R*)-**105** (Scheme 27) [78]. Application of other organometallics paved the way to synthesis of a variety of 3-substituted 2-amino-1,3-propanodiols.

#### 1,3-Diamino-2-hydroxy derivatives

**Scheme 28:**
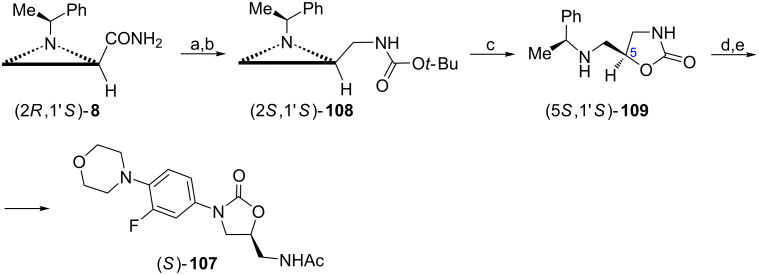
Synthesis of (*S*)-linezolid (*S*)-**107**. Reagents and conditions: a) LiAlH_4_, THF, 0 °C to reflux; b) Boc_2_O, MeOH, rt, overnight; c) BF_3_·OEt_2_, THF, 0 °C to reflux, 2 h; d) 1-bromo-3-fluoro-4-morpholinobenzene, CuI, K_2_CO_3_, MeNHCH_2_CH_2_NHMe, toluene, 115 °C, 27 h; e) HCOONH_4_, 10% Pd/C, MeOH, overnight then AcCl, Et_3_N, CH_2_Cl_2_, rt, overnight.

Linezolid ((*S*)-**107**) represents a new class of 1,3-oxazolidin-2-one antibiotics and contains the (2*S*)-1,3-diamino-2-hydroxypropyl fragment which could also be derived from the aziridine amide (2*R*,1'*S*)-**8** (Scheme 28) [19]. *N*-Boc-protected amine (2*S*,1'*S*)-**108** was converted to a key 1,3-oxazolidin-2-one (5*S*,1'*S*)-**109** by trifluoroborate-catalyzed regioselective and stereospecific (S_N_2 displacement) cyclization. *N*-Arylation and debenzylation followed by acylation provided enantiomerically pure (*S*)-linezolid ((*S*)-**107**).

#### 2-Amino-1,3,4-triols

**Scheme 29:**
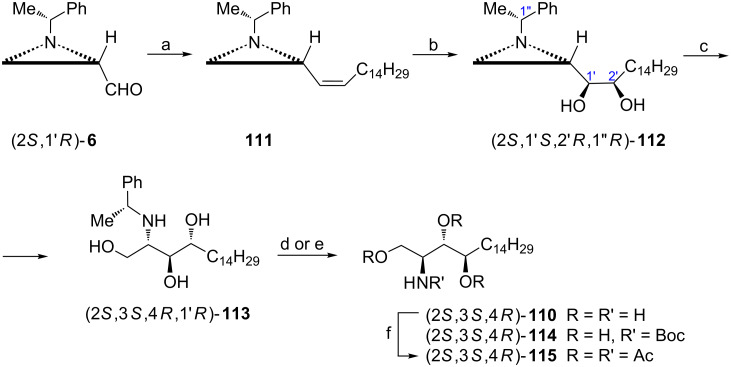
Synthesis of (2*S*,3*S*,4*R*)-2-aminooctadecane-1,3,4-triol (ᴅ-*ribo*-phytosphingosine) (2*S*,3*S*,4*R*)-**110**. Reagents and conditions: a) Ph_3_P^+^CH_2_C_14_H_29_ Brˉ, LiHMDS, THF, −78 °C to rt, 2 h; b) OsO_4_, NMO, Me_2_O/H_2_O, −10 °C, 10 h; c) AcOH, CH_2_Cl_2_, rt, 8 h then KOH, EtOH, rt, 3 h; d) H_2_, Pd(OH)_2_, EtOH, 100 psi, rt; e) H_2_, Pd(OH)_2_, Boc_2_O, EtOH, rt, 100 psi; f) Ac_2_O, pyridine, rt, overnight.

ᴅ-*ribo*-Phytosphingosine ((2*S*,3*S*,4*R*)-2-aminooctadecane-1,3,4-triol, (2*S*,3*S*,4*R*)-**110**) appears to be the most common from other stereoisomeric phytosphingosines which are present in many species and show diversified biological activity. From several synthetic approaches to ᴅ-*ribo*-phytosphingosine [79] application of the aziridine aldehyde (2*S*,1'*R*)-**6** provided (2*S*,3*S*,4*R*)-**110** in four steps with full control of stereochemistry (Scheme 29) [80]. Since the 2*S* absolute configuration of the final product was already secured in the starting aldehyde introduction of the two others required *cis*-dihydroxylation of the *Z*-alkene **111**. The highest diastereoselectivity (99:1) was observed at −10 °C providing the aziridine diol (2*S*,1'*S*,2'*R*,1''*R*)-**112** as a major diastereoisomer. The regioselective aziridine ring opening combined with ester hydrolysis and debenzylation gave enantiomerically pure (2*S*,3*S*,4*R*)-**110** and two stable derivatives (2*S*,3*S*,4*R*)-**114** and (2*S*,3*S*,4*R*)-**115** (Scheme 29).

#### Amino acids and derivatives

**Scheme 30:**
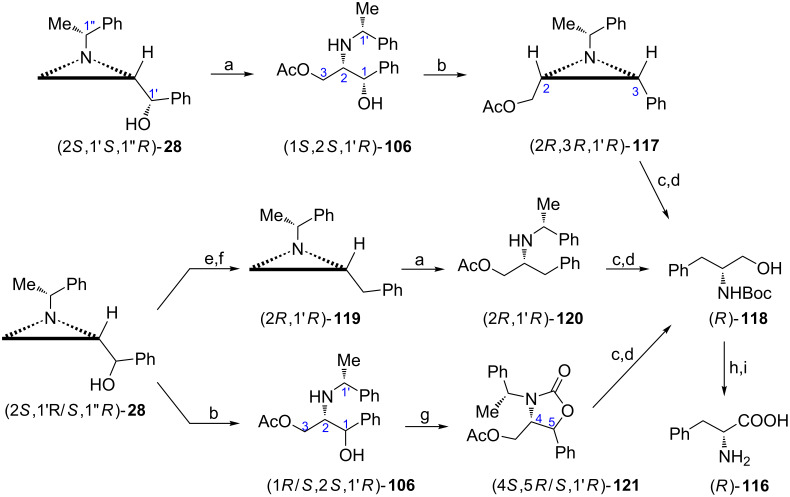
Syntheses of ᴅ-phenylalanine (*R*)-**116**. Reagents and conditions: a) AcOH, CH_2_Cl_2_, reflux, 4 h; b) MsCl, TEA, CH_2_Cl_2_, −78 °C to rt, 6 h; c) H_2_, Pd(OH)_2_, Boc_2_O, MeOH, rt, 7 h; d) KOH, EtOH, rt, 10 min; e) MsCl, TEA, CH_2_Cl_2_, 0 °C, 2 h; f) LiAlH_4_, ether, rt, 4 h; g) CDI, CH_2_Cl_2_, rt, 24 h; h) RuCl_3_, NaIO_4_, CCl_4_/MeCN/H_2_O, rt, 7 h; i) HCl, reflux, 4 h.

**α-Amino acids:** Homochiral amino alcohols of general formula RCH(NH_2_)CH_2_OH as precursors of α-amino acids can be synthesized from aziridine-2-methanols either by functionalization at C3 (Scheme 12 and Scheme 13) or by opening of the aziridine ring to form 2-amino-1,3-diols **12** (Schemes 22–24) combined with the removal of the secondary hydroxy group when simple amino acids (R = alkyl, aryl) are to be prepared. For the latter case the known strategies will be illustrated by syntheses of ᴅ-phenylalanine (*R*)-**116** [81–82]. The acetate (1*S*,2*S*,1'*R*)-**106** obtained from the aziridine alcohol (2*S*,1'*S*,1''*R*)-**28** was subjected to mesylation to yield *cis*-2,3-disubstituted aziridine (2*R*,3*R*,1'*R*)-**117** (Scheme 30). The hydrogenolytic cleavage of the aziridine ring occurred regiospecifically at the N**–**C3 bond (benzylic position) to provide *N*-Boc-ᴅ-phenylalaninol ((*R*)-**118**) after saponification. When mesylation of (2*S*,1'*R*/*S*,1''*R*)-**28** was followed by LiAlH_4_ reduction the aziridine (2*R*,1'*R*)-**119** was produced from which the acetate (2*R*,1'*R*)-**120** and next (*R*)-**118** were formed. The aziridine ring opening in (2*S*,1'*R*/*S*,1''*R*)-**28** gave the acetate (1*R*/*S*,2*S*,1'*R*)-**106** which was transformed into the 1,3-oxazolidin-2-one (4*S*,5*R*/*S*,1'*R*)-**121** a precursor to (*R*)-**118**. Catalytic ruthenium tetroxide oxidation of (*R*)-**118** followed by hydrolysis gave ᴅ-phenylalanine (*R*)-**116** as the hydrochloride salt [83]. Other α-amino acids of general formula RCH_2_CH(NH_2_)COOH, e.g., R = Me, iPr, PhCH_2_, were prepared in a similar way [76].

**Scheme 31:**
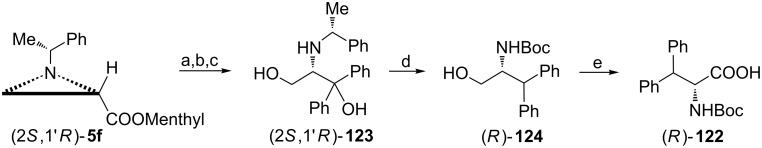
Synthesis of *N*-Boc-ᴅ-3,3-diphenylalanine ((*R*)-**122**). Reagents and conditions: a) PhMgBr, THF, −78 °C, 2 h; b) AcOH, CH_2_Cl_2_, rt, 4 h; c) KOH, EtOH, rt, 2 h; d) H_2_, 20% Pd(OH)_2_/C, HCOOH, 100 psi, rt, 6 h then Boc_2_O, NaOH, AcOEt, rt, 4 h; e) Jones reagent (CrO_3_, H_2_SO_4_, Me_2_CO/H_2_O), 0 °C, 4 h.

A straightforward synthesis of *N*-Boc-ᴅ-3,3-diphenylalanine ((*R*)-**122**) was carried out from the aziridine menthyl ester (2*S*,1'*R*)-**5f** [84]. Phenyl groups were introduced by Grignard reaction while the hydroxymethyl fragment was derived from the aziridine ring opening to produce (2*S*,1'*R*)-**123**. *N*-Debenzylation was accompanied with deoxygenation at the hydroxydiphenylmethyl site to give *N*-Boc-3,3-diphenylalaninol ((*R*)-**124**) which was oxidized with Jones reagent to enantiomerically pure (*R*)-**122** (Scheme 31) useful in pseudopeptide synthesis.

**Scheme 32:**
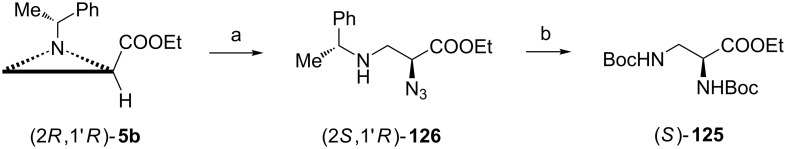
Synthesis of ethyl *N*,*N’*-di-Boc-ʟ-2,3-diaminopropanoate ((*S*)-**125**). Reagents and conditions: a) NaN_3_, AlCl_3_, EtOH/H_2_O, pH 4, rt; b) H_2_, Pd/C, Boc_2_O, rt.

Derivatives of nonproteinogenic ʟ-2,3-diaminopropanoic acid, e.g., (*S*)-**125** were synthesized from the aziridine ester (2*R*,1'*R*)-**5b** employing the aluminum chloride catalyzed opening of the aziridine ring with azide (Scheme 32) [85]. The reaction involved a nucleophilic displacement at more hindered C2 with inversion of configuration to form the azido ester (2*S*,1'*R*)-**126** which was transformed into (*S*)-**125** in usual way. However, since amines can be obtained from azides by a Staudinger reaction the ester (2*S*,1'*R*)-**126** can serve as the starting material to a variety of orthogonally protected derivatives of 2,3-diaminopropanoic acid.

**Scheme 33:**
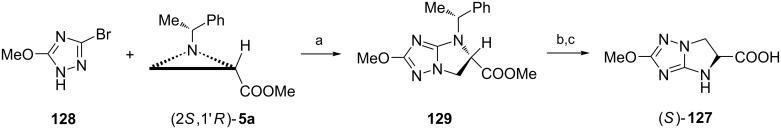
Synthesis of the bicyclic amino acid (*S*)-(+)-**127**. Reagents and conditions: a) BF_3_·OEt_2_, THF, 60 °C, 3 h; b) KOH, MeOH/H_2_O, rt, 1 h; c) H_2_, 10% Pd/C, dioxane, 35 °C, 5 bar, 13 h.

The bicyclic amino acid (*S*)-**127** is a major metabolite of isazofos in corn grain and for toxicological studies both enantiomers were required [17]. To this end the aziridine ester (2*S*,1′*R*)-**5a** was reacted with 3-bromo-5-methoxy-1*H*-1,2,4-triazole (**128**) to give *N*-protected bicyclic amino ester **129** which was next converted into (*S*)-(+)-**127** in two standard steps (Scheme 33) [17]. Its enantiomer was prepared from (2*R*,1′*S*)-**5a**.

**Scheme 34:**
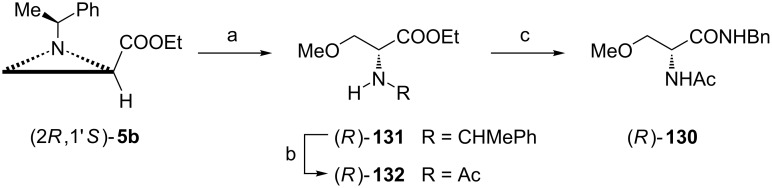
Synthesis of lacosamide, (*R*)-2-acetamido-*N*-benzyl-3-methoxypropanamide (*R*)-**130**. Reagents and conditions: a) MeOH, BF_3_·OEt_2_, MeCN, 90 °C, 3 h; b) H_2_, 20% Pd(OH)_2_/C, Ac_2_O, EtOH, rt, 14 h; c) BnNH_2_, Me_3_Al, CH_2_Cl_2_, rt, 3 h.

Lacosamide ((*R*)-**130**) is a derivative of ᴅ-serine and has found application as an anticonvulsant medication [86]. Under optimized conditions the aziridine ring opening in the ester (2*R*,1'*S*)-**5b** with methanol gave a 94:6 mixture of regioisomeric methoxy amino esters with formation of (*R*)-**131** as a major product (Scheme 34). Catalytic hydrogenation of this mixture in the presence of acetic anhydride produced a crude acetamido ester (*R*)-**132** which was transformed into the final *N*-benzyl amide (*R*)-**130** with of 99.9% ee after crystallization.

**Scheme 35:**
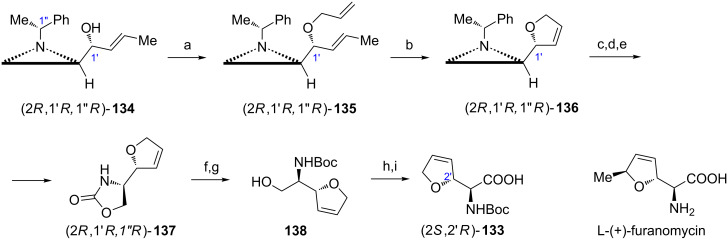
Synthesis of *N*-Boc-norfuranomycin ((2*S*,2'*R*)-**133**). Reagents and conditions: a) H_2_C=CHCH_2_I, NaH, THF, 0 °C to rt, 3 h; b) Grubbs' 1st, CH_2_Cl_2_, rt, 20 h; c) H_2_O, BF_3_·OEt_2_, MeCN, reflux, 3 h; d) CDI, DBU, CH_2_Cl_2_, rt, 12 h; e) Na, NH_3_ liquid, THF, −78 °C, 0.5 h; f) KOH, EtOH/H_2_O, reflux, 2 h; g) Boc_2_O, MeOH, rt, 3 h; h) Dess–Martin periodinane, CH_2_Cl_2_, rt, 1 h; i) NaClO_2_, *t*-BuOH/H_2_O, Me_2_C=CHMe, NaH_2_PO_4_, 0 °C to rt.

ʟ-(+)-Furanomycin (Scheme 35) is a natural nonproteinogenic amino acid which showed pronounced antibacterial activity. From several syntheses of this compound the general approach which employs the aziridine ester (2*R*,1'*R*)-**5b** as a starting material allows to also obtain its 5'-epimer and norfuranomycin (2*S*,2'*R*)-**133** [87]. To construct the 2,5-dihydrofurane ring the aziridine alcohol (2*R*,1'*R*,1''*R*)-**134** (Scheme 35) [88] was converted to the allyl ether (2*R*,1'*R*,1''*R*)-**135** which in the presence of Grubbs 1st generation catalyst produced (2*R*,1'*R*,1''*R*)-**136**. Transformation of the aziridine portion of **136** into an amino acid fragment of norfuranomycin (2*S*,2'*R*)-**133** was accomplished starting from the hydrolytic opening of the aziridine ring and was followed by protection of the amino alcohol and Birch debenzylation to give **137**. After basic hydrolysis and *N*-protection to form **138** a two-step hydroxymethyl to carboxyl oxidation was performed to yield *N*-Boc-norfuranomycin ((2*S*,2'*R*)-**133**). Installation of the 1-methylprop-2-en-1-yl group in (2*R*,1'*R*,1''*R*)-**135** instead of the allyl residue opened the way to synthesis of ʟ-(+)-furanomycin and its 5'-epimer.

**Scheme 36:**
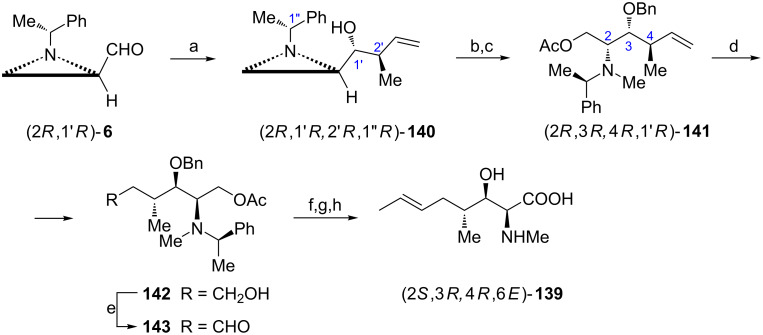
Synthesis of MeBmt (2*S*,3*R*,4*R*,6*E*)-**139**. Reagents and conditions: a) diisopropyl (*S*,*S*)-tartrate (*E*)-crotylboronate, toluene, MS 4 Å, −78 °C, 3 h; b) BnBr, NaH, THF, 0 °C to rt, 3 h; c) TfOMe, MeCN, rt, 10 min then AcOCs, rt, 1 h; d) catecholborane, (Ph_3_P)_3_RhCl, THF, 0 °C, 3 h then NaOH, MeOH, H_2_O_2_, rt, 1 h; e) Swern oxidation; f) 5-(ethylsulfonyl)-1-phenyl-1*H*-tetrazole, KHMDS, DME, −78 °C, 40 min; g) PDC, DMF, rt, 15 h; h) Na, NH_3_ liquid/THF, −78 °C, 15 min.

MeBmt (2*S*,3*R*,4*R*,6*E*)-**139** is a nonproteinogenic amino acid found as a constituent of the naturally occurring cyclic peptide cyclosporine currently in medical use as immunosuppressant [89]. Syntheses of MeBmt are rather challenging endeavor since these amino acids in addition to an *E*-configured C=C bond has three neighboring stereogenic centers. The starting aziridine aldehyde (2*R*,1'*R*)-**6** already introduces the required configuration at C2 and the two other centers of chirality were created by the stereospecific crotylation with a homochiral boronate to give the aziridine alcohol (2*R*,1'*R*,2'*R*,1''*R*)-**140** (Scheme 36) [90]. After *O*-benzylation and *N*-methylation the ring opening in the intermediate aziridinium ion was tried. It appeared that the best regioselectivity (87:13) was achieved with cesium acetate and the major product (2*R*,3*R*,4*R*,1'*R*)-**141** was separated chromatographically. To install two lacking carbon atoms the vinyl moiety was transformed into the respective aldehyde **143** (via a primary alcohol **142**) which when subjected to Julia–Kocienski reaction furnished the *E*-olefinic terminus. Since under these conditions the acetate function was also hydrolyzed the carboxy group was formed by oxidation of the hydroxymethyl residue. To complete the synthesis *N*- and *O*-benzylic protecting groups were removed during the Birch reaction.

**Scheme 37:**
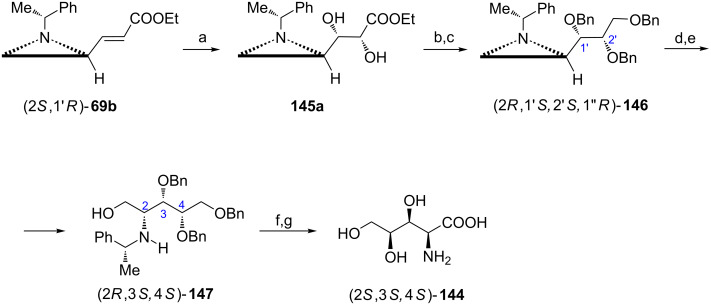
Synthesis of (+)-polyoxamic acid (2*S*,3*S*,4*S*)-**144**. Reagents and conditions: a) AD-mix-α, MeSO_2_NH_2_, *t*-BuOH/H_2_O, 0 °C, 36 h; b) LiAlH_4_, THF, 0 °C, 20 min; c) BnBr, NaH, TBAI, THF, rt, 12 h; d) AcOH, CH_2_Cl_2_, rt, 18 h; e) KOH, EtOH, rt, 2 h; f) Jones reagent, Me_2_O/H_2_O, 0 °C, 4 h; g) H_2_, Pd/C, MeOH, rt, 12 h.

A polyoxamic structural framework was found in polyoxins, natural peptidyl nucleosides with primarily antifungal properties. The stereoselective synthesis of (+)-polyoxamic acid ((2*S*,3*S*,4*S*)-**144**) was successfully carried out starting with Horner–Wadsworth–Emmons olefination of the aziridine aldehyde (2*R*,1'*R*)-**6** which provided a 98:2 mixture of *trans*- and *cis*-acrylates **69b** (Scheme 37) [32]. The major product (2*S*,1'*R*)-**69b** was subjected to Sharpless asymmetric dihydroxylation in the presence of AD-mix-α to give the diol **145a** as a major (10:1) diastereoisomer. The ester moiety in **145a** was reduced and hydroxy groups were protected to give the tribenzyloxy aziridine (2*R*,1'*S*,2'*S*,1''*R*)-**146**. After treatment with acetic acid and ester hydrolysis the intermediary amino alcohol (2*R*,3*S*,4*S*)-**147** was oxidized and hydrogenolytic removal of benzyl groups completed the synthesis.

**Scheme 38:**
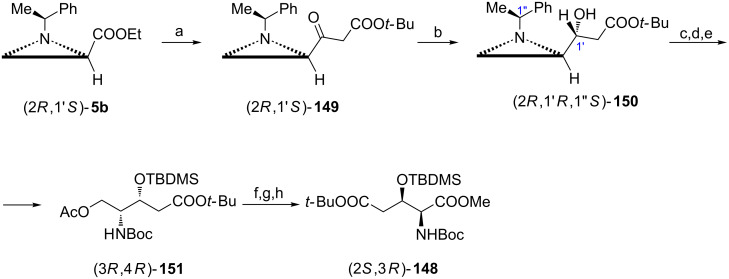
Synthesis of the protected 3-hydroxy-ʟ-glutamic acid (2*S*,3*R*)-**148**. Reagents and conditions: a) LiHMDS, AcO*t*-Bu, THF, −78 °C, 0.5 h; b) NaBH_4_, iPrOH, −40 °C, 1 h; c) TBDMSCl, TEA, DMAP, CH_2_Cl_2_, rt, 17 h; d) AcOH, CH_2_Cl_2_, rt, 10 h; e) H_2_, Pd(OH)_2_, Boc_2_O, MeOH, rt, 3 h; f) KOH, EtOH, 0 °C, 10 min; g) NaIO_4_, RuCl_3_, CCl_4_/MeCN/H_2_O, rt, 7 h; h) MeI, KHCO_3_, DMF, rt, 5 h.

The orthogonally protected 3-hydroxy-ʟ-glutamic acid (2*S*,3*R*)-**148** was obtained from the aziridine ester (2*R*,1′*S*)-**5b** (Scheme 38) [91]. A two-carbon fragment came from *tert*-butyl acetate while the required *R* configuration at C3 in the final product was secured by stereoselective reduction (10:1) of the ketone (2*R*,1′*S*)-**149** to give the aziridine alcohol (2*R*,1′*R*,1''*S*)-**150** as the major product. Silylation of the hydroxy group preceded the aziridine ring opening with acetic acid while hydrogenation in the presence of Boc_2_O led to the formation of (3*R*,4*R*)-**151**. To conclude the synthesis of (2*S*,3*R*)-**148** the hydroxymethyl group was recovered after basic deacetylation and it was further oxidized and esterified. The same methodology was applied in the preparation of (2*S*,3*S*)-**148**.

**Scheme 39:**
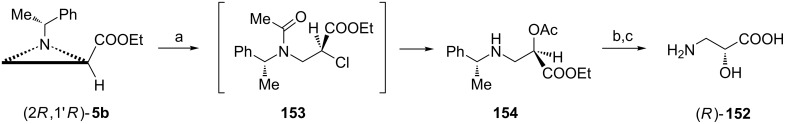
Synthesis of (+)-isoserine (*R*)-**152**. Reagents and conditions: a) AcCl, MeCN, rt, 0.5 h then Na_2_CO_3_, CH_2_Cl_2_/H_2_O, rt; b) H_2_, Pd/C, Boc_2_O, MeOH, rt, 12 h; c) HCl, MeOH, reflux, 1 h then Amberlite IR-120 H.

**β-Amino acids:** (+)-Isoserine ((*R*)-**152**) was synthesized from the aziridine ester (2*R*,1'*R*)-**5b** in three simple steps (Scheme 39) [92]. Treatment of (2*R*,1'*R*)-**5b** with acetyl chloride led to *N*-acetylation with concomitant opening of the corresponding aziridinium ion with the chloride anion at C2 to give an unstable intermediate **153** which in the presence of aqueous base was transformed into the protected ethyl (*R*)-isoserinate **154**. *N*-Debenzylation and hydrolysis completed the synthesis of (+)-isoserine ((*R*)-**152**).

**Scheme 40:**
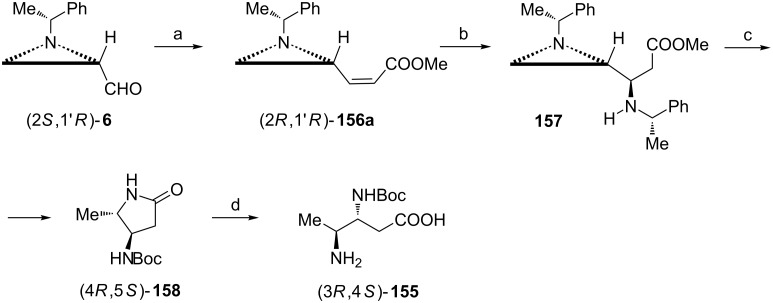
Synthesis of (3*R*,4*S*)-*N*^3^-Boc-3,4-diaminopentanoic acid (3*R*,4*S*)-**155**. Reagents and conditions: a) Ph_3_P=CHCOOMe, MeOH, 0 °C, 1 h; b) (*S*)-PhMeCHNH_2_, MeOH, 50 °C, 7 h; c) H_2_, Pd(OH)_2_, MeOH, rt, 4 h then Boc_2_O, MeOH, rt, 4 h; d) LiOH, MeOH/H_2_O, reflux, 6 h then Dowex 50W X2-200.

Enantiomerically pure (3*R*,4*S*)-*N*^3^-Boc-3,4-diaminopentanoic acid ((3*R*,4*S*)-**155**) was synthesized from the *Z*-acrylate (2*R*,1'*R*)-**156a** prepared in a highly (88:12) stereoselective olefination of the aziridine aldehyde (2*S*,1'*R*)-**6** (Scheme 40) [60]. Michael addition of (*S*)-1-phenylethylamine to **156a** gave almost pure (>99:1) β-amino ester **157** which when subjected to the reductive opening of the aziridine ring was transformed into the pyrrolidin-2-one (4*R*,5*S*)-**158**. Basic hydrolysis produced (3*R*,4*S*)-**155**.

**Scheme 41:**
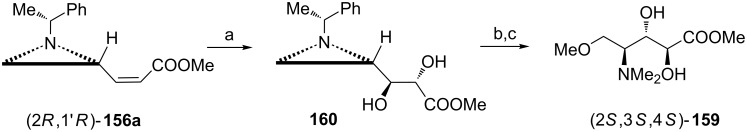
Synthesis of methyl (2*S*,3*S*,4*S*)-4-(dimethylamino)-2,3-dihydroxy-5-methoxypentanoate (2*S*,3*S*,4*S*)-**159**. Reagents and conditions: a) OsO_4_, NMO, acetone, 0 °C to rt, 5 h; b) MeOH, BF_3_·OEt_2_, reflux, 3 h; c) H_2_, HCHO, 10% Pd/C, MeOH, rt, 12 h.

**Other amino acids:** Calyculins were isolated from marine sponges and they are of interest because of possible applications as protein phosphatase 1 and 2A inhibitors [93]. An interesting structural feature of calyculins is a C33–C37 fragment γ-amino acid (2*S*,3*S*,4*S*)-**159**. Its stereocontrolled synthesis involved *cis*-dihydroxylation of the aziridine *cis*-acrylate (2*R*,1'*R*)-**156a** which led to the formation of a 91:9 mixture of diastereoisomeric diols with **160** preponderating (Scheme 41) [34]. The regioselective aziridine ring opening with methanol and catalytic hydrogenation in the presence of formalin gave the final product (2*S*,3*S*,4*S*)-**159**.

**Scheme 42:**
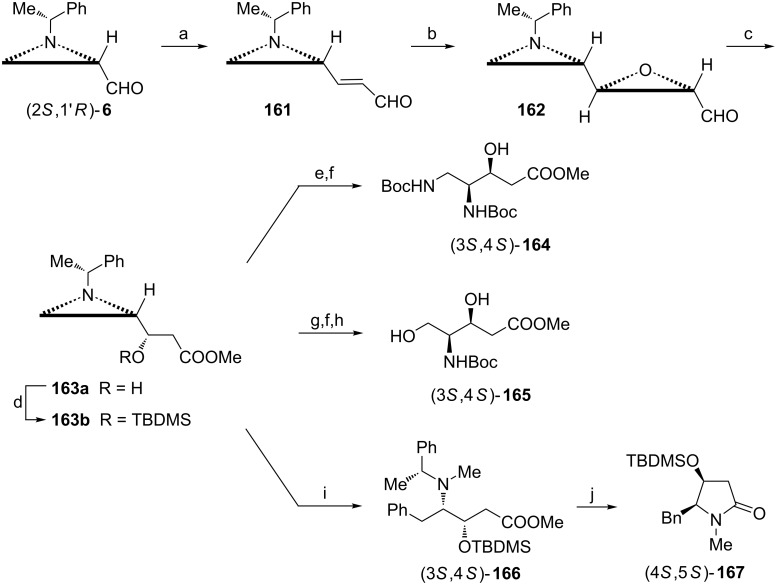
Syntheses of methyl (3*S*,4*S*) 4,5-di-*N*-Boc-amino-3-hydroxypentanoate ((3*S*,4*S*)-**164**), methyl (3*S*,4*S*)-4-*N*-Boc-amino-3,5-dihydroxypentanoate ((3*S*,4*S*)-**165**) and (4*S*,5*S*)-5-benzyl-4-hydroxy-1-methylpyrrolidin-2-one (4*S*,5*S*)-**167**. Reagents and conditions: a) Ph_3_P=CHCHO, toluene, 60 °C, 18 h; b) 35% H_2_O_2_, (*S*)-[diphenyl(trimethylsilyloxy)methyl]pyrrolidine, EtOH, rt, 6 h; c) 3-benzyl-4,5-dimethylthiazolium chloride, DIPEA, MeOH, CH_2_Cl_2_, rt, 24 h; d) TBDMSCl, DMAP, CH_2_Cl_2_, 0 °C to rt, 6 h; e) NaN_3_, BF_3_·OEt_2_, MeCN, 50 °C, 6 h; f) H_2_, 50% Pd(OH)_2_/C, Boc_2_O, MeOH, rt, 6 h; g) AcOH neat, rt, 6 h; h) K_2_CO_3_, MeOH, rt, 0.5 h then HF•pyridine, MeCN, 0 °C, 0.5 h; i) TfOMe, dioxane, 0 °C, 10 min then CuI, PhMgBr, THF, 0 °C, 10 min; j) H_2_, 50% Pd(OH)_2_/C, MeOH, rt, 6 h.

**Scheme 43:**
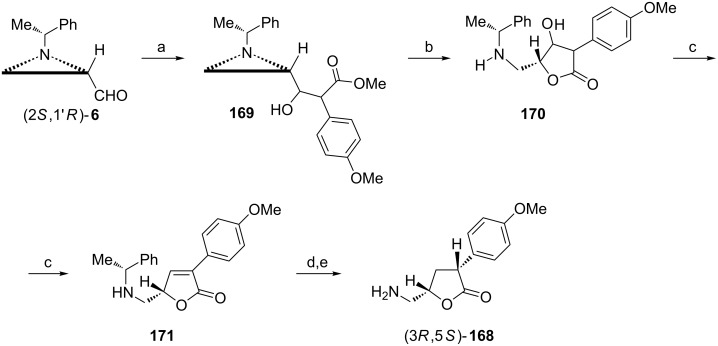
Syntheses of (3*R*,5*S*)-5-(aminomethyl)-3-(4-methoxyphenyl)dihydrofuran-2(3*H*)-one ((3*R*,5*S*)-**168**). Reagents and conditions: a) 4-MeOC_6_H_4_CH_2_COOMe, LiHMDS, THF, −78 °C, 0.5 h; b) BF_3_·OEt_2_, MeCN, reflux, 2 h; c) H_2_SO_4_, THF, 70 °C, 48 h; d) H_2_, Pd(OH)_2_, Boc_2_O, EtOH, 1 h; e) HCl/MeOH, rt, 12 h.

The innovative application of the aldehyde (2*S*,1'*R*)-**6** in syntheses of nonproteinogenic γ-amino hydroxy acids and their cyclic forms (pyrrolidin-2-ones) takes advantage of the stereoselective epoxidation of the aziridine acrylaldehyde **161** to predominantly (98:2) form the aziridine epoxide **162** when (*S*)-[diphenyl(trimethylsilyloxy)methyl]pyrrolidine was used as a catalyst (Scheme 42) [94]. A key β-hydroxyester **163** was produced from the epoxide **162** employing *N*-heterocyclic carbene catalysis. Openings of the aziridine ring in **163a** with azide or in **163b** with in acetic acid provided enantiomerically pure methyl (3*S*,4*S*)-4,5-di-*N*-Boc-amino-3-hydroxypentanoate **164** or 4-*N*-Boc-amino-3,5-dihydroxypentanoate **165**, respectively. The latter compound as an unprotected acid was identified as a component of immunosuppressive thalassospiramide A [95] and siderophores called crochelins [96]. On the other hand, the former one was used in the synthesis of edenine A and D analogues [97] to study their biological properties. When the *O*-protected ester **163b** was subjected to methylation and the corresponding aziridinium ion was treated with phenylmagnesium bromide a regioselective opening of the aziridine ring occurred at the less substituted carbon atom to give the protected pentanoate (3*S*,4*S*)-**166**. After catalytic debenzylation it was transformed into a silylated (4*S*,5*S*)-5-benzyl-4-hydroxy-1-methylpyrrolidin-2-one **167**, a molecule having a structural core of antifungal (+)-preussin [98].

(3*R*,5*S*)-5-(Aminomethyl)-3-(4-methoxyphenyl)dihydrofuran-2(3*H*)-one ((3*R*,5*S*)-**168**) was discovered as a potential medication in Parkinson’s disease [99]. Synthesis of enantiomerically pure (3*R*,5*S*)-**168** could be accomplished from the aziridine aldehyde (2*S*,1'*R*)-**6** because the butyrolactone ring formation would proceed with inversion of configuration at C2 in the aziridine ring (Scheme 43) [100]. Thus, condensation of the aldehyde (2*S*,1'*R*)-**6** with lithium enolate of methyl 4-methoxyphenylacetate would give the β-hydroxyester **169** which when treated with Lewis acid experienced the aziridine ring cleavage with simultaneous dihydrofuran-2-one ring closure to produce **170** contaminated with small amounts of the corresponding furan-2(5*H*)-one **171**. Dehydration of **170** was completed in the presence of acid. Catalytic hydrogenation of the C=C bond in **171** took place preferentially (9:1) from the less hindered side to finally give (3*R*,5*S*)-**168** as the hydrochloride salt.

**Scheme 44:**
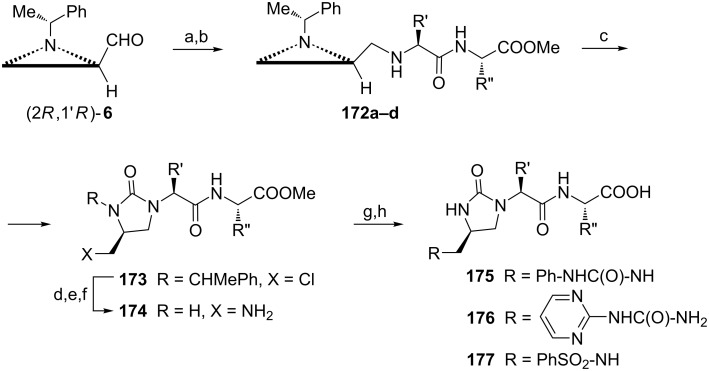
Syntheses of a series of imidazolin-2-one dipeptides **175**–**177** (for R' and R'' see text). Reagents and conditions: a) H_2_NCHR'C(O)NHCHR''COOMe, MgSO_4_, CH_2_Cl_2_, rt, 5 h; b) NaBH_3_CN, MeOH, rt, overnight; c, triphosgene, NaH, THF, −10 °C, 2 h; d) MsOH, anisole, hexane, reflux, 4 h; e) NaN_3_, DMF, 80 °C, overnight; f) H_2_, Pd/C, MeOH, rt, overnight; g) PhNCO, THF, rt, 3 h or phenyl (pyrimidin-2-yl)carbamate, MeCN, reflux, 3 h or PhSO_2_Cl, TEA, THF, reflux, 1 h; h) NaOH 1N, MeOH/H_2_O, rt, 1 h.

Substituted imidazolin-2-ones are of interest as potential aminoacyl-tRNA synthase inhibitors. When the aziridine-aldehyde (2*R*,1'*R*)-**6** was subjected to the reductive amination with 4 dipeptides secondary amines **172** (**a** R' = iBu, R''= *sec*-Bu; **b** R' = R'' = iBu; **c** R' = *sec*-Bu, R'' = iBu; **d** R' = R'' = *sec*-Bu) were produced (Scheme 44) [101]. In the presence of triphosgene a series of imidazolin-2-ones having a 2-chloromethyl substituent **173** was formed. Removal of the 1-phenylethyl moiety and a subsequent replacement of the chlorine atom by the amino group via azide gave imidazolin-2-one dipeptides **174** which were transformed into 12 derivatives **175**–**177** after hydrolysis of the corresponding methyl esters. Although docking simulation predicted binding of these compounds to isoleucyl-tRNA synthetase (IleRS) none of them showed inhibitory activity.

#### Alkaloids

**Scheme 45:**
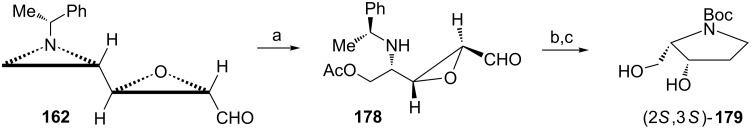
Syntheses of (2*S*,3*S*)-*N*-Boc-3-hydroxy-2-hydroxymethylpyrrolidine ((2*S*,3*S*)-**179**). Reagents and conditions: a) AcOH, CH_2_Cl_2_, rt, 6 h; b) H_2_, 50% Pd(OH)_2_/C, Boc_2_O, MeOH, rt, 12 h; c) KOH, EtOH, rt, 0.5 h.

**Pyrrolidines:** Pyrrolidine alkaloids and among them polyhydroxypyrrolidines like 1,4-dideoxy-1,4-imino-ʟ-ribitol (2*S*,3*S*,4*R*)-**182** were found as components of a variety of plants and exhibit a wide range of biological properties including inhibition of glucosidases [102]. When the aziridine epoxide **162** was treated with acetic acid the aziridine ring cleavage was observed to give the epoxyaldehyde **178** (Scheme 45) [94]. Catalytic hydrogenation followed by saponification converted **178** into (2*S*,3*S*)-*N*-Boc-3-hydroxy-2-hydroxymethylpyrrolidine (2*S*,3*S*)-**179**, one of the simplest members of the iminosugar family.

**Scheme 46:**
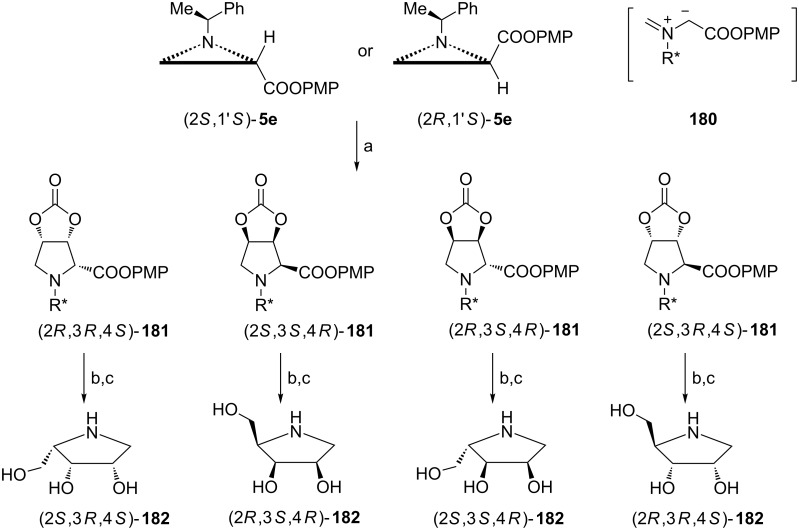
Syntheses of enantiomers of 1,4-dideoxy-1,4-imino-ʟ- and -ᴅ-lyxitols (2*S*,3*R*,4*S*)-**182** and (2*R*,3*S*,4*R*)-**182**, and 1,4-dideoxy-1,4-imino-ʟ- and -ᴅ-ribitol (2*S*,3*S*,4*R*)-**182** and (2*R*,3*R*,4*S*)-**182**. Reagents and conditions: a) vinylene carbonate, toluene, 280 °C, 0.5 h; b) LiAlH_4_, THF, rt; c) H_2_, Pd(OH)_2_, HCl, MeOH/H_2_O, rt.

Reaction of aziridine 4-methoxyphenyl esters either (2*R*,1′*S*)-**5e** or (2*S*,1′*S*)-**5e** with vinylene carbonate at 280 °C gave a mixture of four stereoisomers with low (ca. 3:1) diastereoselectivity and in a non-enantioselective manner (Scheme 46) [21]. Undoubtedly, it was the 1,3-dipolar cycloaddition of the azomethine ylide **180** with an electron-rich alkene. Fortunately, cycloadducts (2*R*,3*R*,4*S*)-, (2*S*,3*S*,4*R*)-, (2*R*,3*S*,4*R*)- and (2*S*,3*R*,4*S*)-**181** were efficiently separated chromatographically and after reduction and catalytic removal of the chiral auxiliary enantiomerically pure 1,4-dideoxy-1,4-imino-ʟ- and -ᴅ-lyxitols, (2*S*,3*R*,4*S*)-**182** and (2*R*,3*S*,4*R*)-**182**, and 1,4-dideoxy-1,4-imino-ʟ- and -ᴅ-ribitols, (2*S*,3*S*,4*R*)-**182** and (2*R*,3*R*,4*S*)-**182** were obtained as hydrochloride salts.

**Scheme 47:**
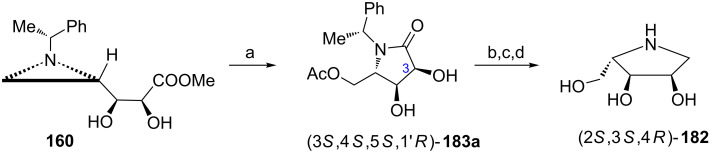
Synthesis of 1,4-dideoxy-1,4-imino-ʟ-ribitol (2*S*,3*S*,4*R*)-**182**. Reagents and conditions: a) AcOH, CH_2_Cl_2_, reflux, 3 h; b) BH_3_•SMe_2_, THF, 0 °C, 10 h; c) H_2_, Pd(OH)_2_, MeOH, 50 psi, rt, 12 h; d) HCl, MeOH, 0 °C, 5 h.

Since the pyrrolidine (2*S*,3*S*,4*R*)-**182** has three stereogenic centers of the same configuration as in (2*S*,3*S*,4*S*)-**159** its synthesis started from the common intermediate diol **160** (Scheme 41) with the aziridine ring opening to produce the pyrrolidin-2-one (3*S*,4*S*,5*S*,1'*R*)-**183a** (Scheme 47) [34]. The amide bond reduction and *N*-debenzylation gave 1,4-dideoxy-1,4-imino-ʟ-ribitol (2*S*,3*S*,4*R*)-**182** which was isolated as the hydrochloride salt.

**Scheme 48:**
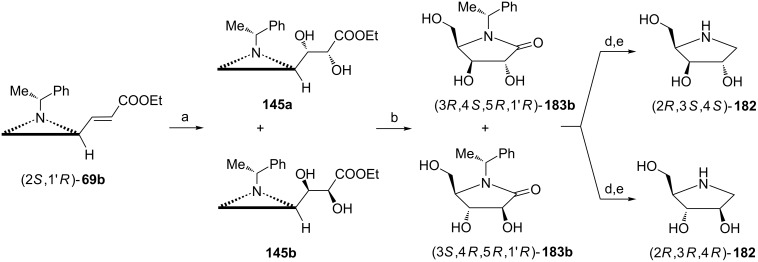
Syntheses of 1,4-dideoxy-1,4-imino-ᴅ-arabinitol (2*R*,3*R*,4*R*)-**182** and 1,4-dideoxy-1,4-imino-ᴅ-xylitol (2*R*,3*S*,4*S*)-**182**. Reagents and conditions: a) OsO_4_, NMO, THF/H_2_O, rt, 12 h; b) AcOH, CH_2_Cl_2_, rt, 18 h, then toluene, 50 °C, 12 h; c) separation of diastereoisomers; d) BH_3_·SMe_2_, THF, 0 °C to rt, 12 h, e) H_2_, Pd(OH)_2_, MeOH, 3 h.

Syntheses of 1,4-dideoxy-1,4-imino-ᴅ-arabinitol ((2*R*,3*R*,4*R*)-**182**) and 1,4-dideoxy-1,4-imino-ᴅ-xylitol ((2*R*,3*S*,4*S*)-**182**) were accomplished starting from the aziridine (*E*)-acrylate (2*S*,1'*R*)-**69b** readily prepared from the aldehyde (2*R*,1'*R*)-**6** (Scheme 48) [31]. The (*E*)-acrylate was subjected to *cis*-dihydroxylation to give a 1:1 mixture of the diastereoisomeric diols **145a** and **145b** [103]. Their separation could be achieved after transformation into pyrrolidin-2-ones (3*S*,4*R*,5*R*,1'*R*)-**183b** (by crystallization from cold ethanol) and (3*R*,4*S*,5*R*,1'*R*)-**183b** (by flash chromatography). After reduction and hydrogenolysis they were converted into 1,4-dideoxy-1,4-imino-ᴅ-arabinitol and ((2*R*,3*R*,4*R*)-**182**) and 1,4-dideoxy-1,4-imino-ᴅ-xylitol ((2*R*,3*S*,4*S*)-**182**), respectively. Their enantiomers were prepared from the aziridine aldehyde (2*S*,1'*R*)-**6** in an analogous manner.

**Scheme 49:**
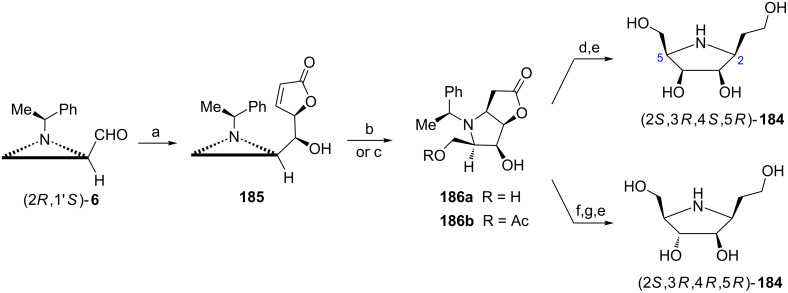
Syntheses of natural 2,5-imino-2,5,6-trideoxy-ʟ-*gulo*-heptitol ((2*S*,3*R*,4*R*,5*R*)-**184**) and its C4 epimer (2*S*,3*R*,4*S*,5*R*)-**184**. Reagents and conditions: a) 2-trimethylsilyloxyfuran, ZnBr_2_, THF, 0 °C, 12 h; b) TFA, THF/H_2_O, rt, 15 h; c) AcOH, CH_2_Cl_2_, rt, 15 h; d) BH_3_·SMe_2_, THF, rt, 4 h; e) H_2_, Pd(OH)_2_/C, MeOH, rt, 7 h; f) Ph_3_P, DIAD, 4-O_2_NC_6_H_4_COOH, toluene, 100 °C, 5 h; g) BH_3_·SMe_2_, THF, 50 °C, 4 h.

2,5-Imino-2,5,6-trideoxy-ʟ-*gulo*-heptitol ((2*S*,3*R*,4*R*,5*R*)-**184**) an iminosugar isolated from *Hyacintus orientalis* was recognized as inhibitor of glycosidases [104]. The efficient syntheses of (2*S*,3*R*,4*R*,5*R*)-**184** and its C4 epimer (2*S*,3*R*,4*S*,5*R*)-**184** were accomplished from the aziridine aldehyde (2*R*,1'*S*)-**6** (Scheme 49) [105]. The Lewis acid-catalyzed nucleophilic *anti-*addition of 2-trimethylsilyloxyfuran to (2*R*,1'*S*)-**6** furnished diastereoisomerically pure secondary alcohol **185** since chelation-controlled transition state was involved. Acid-induced aziridine ring openings and subsequent conjugate additions to the α,β-unsaturated lactone led to the formation of *cis*-fused [5,5']bicyclic compounds **186a** or **186b**. Reduction of the lactone moiety in **186a** and subsequent deprotection gave (2*S*,3*R*,4*S*,5*R*)-**184**. In order to synthesize the natural (2*S*,3*R*,4*R*,5*R*)-**184** the lactone **186b** was subjected to Mitsunobu reaction followed by the ester reduction and the hydrogenolytic cleavage of the 1-phenylethyl group.

**Scheme 50:**
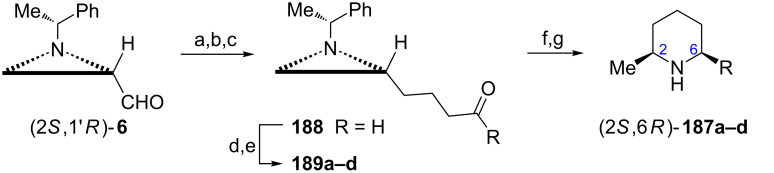
Syntheses of (−)-dihydropinidine ((2*S*,6*R*)-**187a**) (R = C_3_H_7_) and (2*S*,6*R*)-isosolenopsins (2*S*,6*R*)-**187b** (R = C_9_H_19_), (2*S*,6*R*)-**187c** (R = C_11_H_23_) and (2*S*,6*R*)-**187d** (R = C_15_H_31_). Reagents and conditions: a) Ph_3_P^+^(CH_2_)_3_OH Brˉ, BuLi, THF, rt, 12 h; b) 2-O_2_NC_6_H_4_SO_2_NHNH_2_, Et_3_N, rt, 12 h; c) Swern oxidation; d) RMgBr, THF, 0 °C to rt, 1 h; e) Dess–Martin periodinane, CH_2_Cl_2_, 0 °C to rt, 1 h; f) H_2_, 20% Pd(OH)_2_/C, MeOH, rt, 12 h; g) 1 M HCl, MeOH.

**Piperidines:** A *cis*-disubstituted piperidine scaffold was identified in several piperidine alkaloids of diverse biological activities [106]. (−)-Dihydropinidine (2*S*,6*R*)-**187a**, isosolenopsin (2*S*,6*R*)-**187b**, and isosolenopsins A (2*S*,6*R*)-**187c** and B (2*S*,6*R*)-**187d** were synthesized as hydrochloride salts from the aldehyde (2*S*,1'*R*)-**6** applying a reductive amination as a key step (Scheme 50) [33]. To this end the aldehyde (2*S*,1'*R*)-**6** was transformed into its higher homolog (2*R*,1'*R*)-**188** employing Wittig olefination, the C=C bond reduction and Swern oxidation. The required alkyl chains were introduced by Grignard reagents and mixtures of diastereoisomeric alcohols were oxidized to ketones **189a**–**d**. Catalytic hydrogenation allowed for the aziridine ring opening and the removal of the 1-phenylethyl group and also for the reduction of the intermediate cyclic imine which appeared stereospecific.

**Scheme 51:**
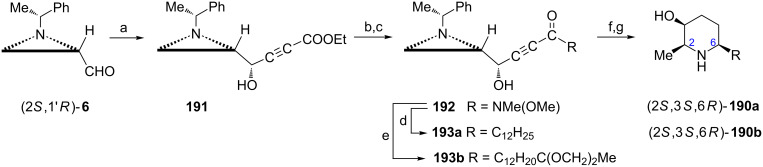
Syntheses of (+)-deoxocassine ((2*S*,3*S*,6*R*)-**190a**, R = C_12_H_25_) and (+)-spectaline ((2*S*,3*S*,6*R*)-**190b**, R = C_12_H_24_C(O)Me). Reagents and conditions: a) HC≡CCOOEt, LiHMDS, THF, −78 °C, 2 h; b) TfOTBDMS, 2,6-lutidine, CH_2_Cl_2_, 0 °C, 10 min.; c) MeONHMe, iPrCl, THF, −10 °C, 1 h; d) C_12_H_25_MgBr, THF, 0 °C to rt, 1 h; e) HC≡C(CH_2_)_10_C(OCH_2_)_2_CH_3_, LiHMDS, THF, −78 °C, 1 h; f) H_2_, 20% Pd(OH)_2_/C, MeOH, rt, 12 h; g) 1 M HCl, THF, rt, 24 h.

**Scheme 52:**
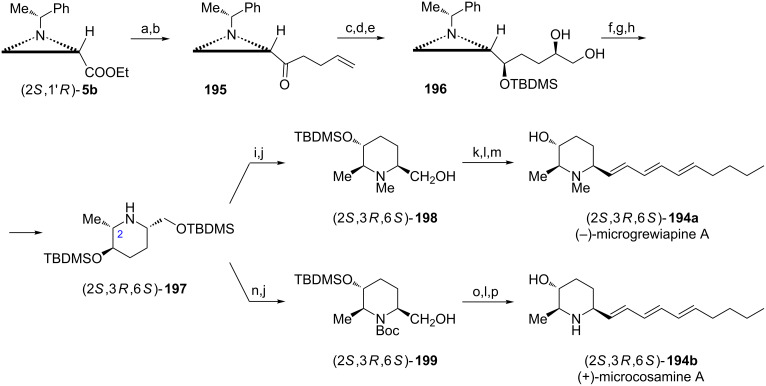
Synthesis of (−)-microgrewiapine A ((2*S*,3*R*,6*S*)-**194a**) and (+)-microcosamine A ((2*S*,3*R*,6*S*)-**194b**). Reagents and conditions: a) MeONHMe, iPrMgCl, THF, −10 °C, 1 h; b) H_2_C=CHCH_2_CH_2_MgBr, THF, 0 °C to rt, 1 h; c) NaBH_4_, ZnCl_2_, MeOH, −78 °C, 1 h; d) TfOTBDMS, lutidine, CH_2_Cl_2_, 0 °C, 0.5 h; e) AD-mix-β, (DHQD)_2_PHAL, MeSO_2_NH_2_, NaHCO_3_, *t*-BuOH/H_2_O, 0 °C, 4 h; f) TBDMSCl, imidazole, CH_2_Cl_2_, rt, 3 h; g) MsCl, TEA, DMAP, rt, 0,5 h; h) H_2_, 20% Pd(OH)_2_/C, MeOH, rt, 12 h; i) 37% HCHO, NaBH_3_CN, AcOH, rt, 1 h; j) HF·pyridine, THF, 0 °C to rt, 12 h; k) Swern oxidation; l) 5-[(2*E*,4*E*)-nona-2,4-dien-1-ylsulfonyl]-1-phenyl-1*H*-tetrazole, KHMDS, 18-crown-6, DME/THF, −78 °C, 2 h and rt overnight; m) TBAF, THF, rt, 1 h; n) Boc_2_O, NaHCO_3_, MeOH, 0 °C to rt, 3 h; o) Dess–Martin periodinane, CH_2_Cl_2_, 0 °C to rt, 4 h; p) 3 M HCl, MeOH, rt, 12 h.

Two other piperidine alkaloids, (+)-deoxocassine ((2*S*,3*S*,6*R*)-**190a**) and (+)-spectaline ((2*S*,3*S*,6*R*)-**190b**) were synthesized as hydrochloride salts in a similar way [33]. However, to introduce the hydroxy group at C3 of the piperidine ring addition of a lithium acetylide to the aziridine aldehyde (2*S*,1'*R*)-**6** was performed (Scheme 51) instead of alkylation (Scheme 50). Two diastereoisomeric acetylenic alcohols were formed in the reaction with lithium ethyl propiolate in an 8:2 ratio, and the major product **191** had the required configuration. After protection of the hydroxy group in **191** Weinreb amide **192** was prepared to facilitate the installation of the respective alkyl chains in ketones **193a** and **193b**. They were transformed into (+)-deoxocassine ((2*S*,3*S*,6*R*)-**190a**) and (+)-spectaline ((2*S*,3*S*,6*R*)-**190b**) as already described.

(+)-Microgrewiapine A ((2*R*,3*S*,6*R*)-**194a**) [107] and (+)-microcosamine A ((2*R*,3*S*,6*R*)-**194b**) [108] have recently been isolated and exhibited interesting biological activities, for example cytotoxicity [107]. The total synthesis of (−)-microgrewiapine A was initiated from the aziridine ketone **195** readily prepared from the ester (2*S*,1'*R*)-**5b** (Scheme 52) [109]. After chelation-controlled reduction of **195** with the NaBH_4_/ZnCl_2_ mixture and protection of a secondary alcohol the asymmetric dihydroxylation of the terminal C=C bond with AD-mix-β provided an inseparable 82:18 mixture of diastereoisomers with the diol **196** as a major product. Silylation of the terminal hydroxy group was followed by mesylation of a secondary one to facilitate the piperidine ring closure triggered by hydrogenolytic removal of the chiral auxiliary to form a *cis*-2,6-disubstituted piperidine framework in (2*S*,3*R*,6*S*)-**197**. *N*-Methylation of (2*S*,3*R*,6*S*)-**197** was accomplished by reductive amination while a selective deprotection provided the hydroxymethyl group in (2*S*,3*R*,6*S*)-**198**. Swern oxidation, Julia–Kocienski olefination and desilylation gave (−)-(2*S*,3*R*,6*S*)-**194a** which appeared to be the enantiomer of natural microgrewiapine A although its structure was identical with the originally proposed. When the total synthesis started from the ester (2*R*,1'*S*)-**5b** and AD-mix-α was used the natural (+)-microgrewiapine A (2*R*,3*S*,6*R*)-**194a** was obtained. To synthesize the (+)-microcosamine A the protected piperidine (2*S*,3*R*,6*S*)-**197** served as a starting material and was first converted into the *N*-Boc derivative while selective desilylation exposed the hydroxymethyl group to give (2*S*,3*R*,6*S*)-**199**. Oxidation to the respective aldehyde was accomplished with Dess–Martin periodinane and olefination was carried out as already described. Acidic hydrolysis of *N*-Boc and *O*-TBDMS groups afforded (2*S*,3*R*,6*S*)-**194b** which was identical with the natural (+)-microcosamine A.

1-Deoxynojirimycin was discovered in several natural species and later found as a potent inhibitor of glycosidases [110]. ʟ-1-Deoxynojirimycin ((2*S*,3*S*,4*S*,5*R*)-**200**) and five of its stereoisomers were synthesized in a unique approach using (2*S*,1'*R*)-**5b** as a starting material [88]. When the aziridine ketone (2*S*,1'*R*)-**201** was treated with a NaBH_4_/ZnCl_2_ mixture the aziridine alcohol (2*S*,1'*R*,1''*R*)-**202** was stereoselectively formed in a chelation-controlled reduction establishing the absolute configurations at C2 and C3 in three final products (2*S*,3*S*,4*S*,5*R*)-**200**, (2*S*,3*S*,4*S*,5*S*)-**200**, (2*S*,3*S*,4*R*,5*S*)-**200**. A triple bond reduction and a protection of the hydroxy group preceded the aziridine ring opening to provide **203** after basic hydrolysis. Installation of the allyl group at the nitrogen atom required prior derivatization as an oxazolidin-2-one and Birch reduction to furnish **204**. To construct a properly functionalized piperidine ring olefin metathesis was performed to supply the key intermediate **205**. Taking advantage of the steric bulkiness of the *tert*-butyldimethylsilyloxy group in **205** three different approaches to stereoselective dihydroxylations of the C=C bond were elaborated. Thus, treatment with Oxone^®^ gave the *anti*-epoxide **206** which was regioselectively opened in a boron trifluoride-catalyzed isopropylidenation to yield **207**. A two-step removal of protecting groups led to the formation of (2*S*,3*S*,4*S*,5*R*)-2-(hydroxymethyl)piperidine-3,4,5-triol ((2*S*,3*S*,4*S*,5*R*)-**200**, ʟ-1-deoxynojirimycin). Direct dihydroxylation of **205** with osmium tetroxide introduced a *cis*-diol moiety oriented anti to the *tert*-butyldimethylsilyloxy group and basic hydrolysis of **208** gave (2*S*,3*S*,4*S*,5*S*)-2-(hydroxymethyl)piperidine-3,4,5-triol ((2*S*,3*S*,4*S*,5*S*)-**200**, ʟ-1-deoxymannojirimycin). After removal of the silyl protection from **205** the epoxidation with MCPBA produced the *syn*-epoxide **209** which was converted into (2*S*,3*S*,4*R*,5*S*)-2-(hydroxymethyl)piperidine-3,4,5-triol ((2*S*,3*S*,4*R*,5*S*)-**200**, ʟ-1-deoxyaltronojirimycin) as described earlier [111].

**Scheme 53:**
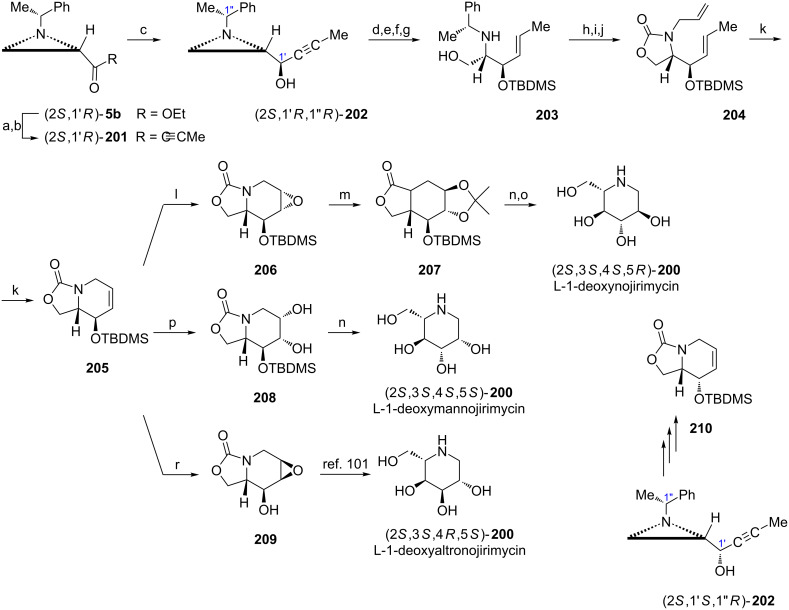
Syntheses of ʟ-1-deoxynojirimycin ((2*S*,3*S*,4*S*,5*R*)-**200**), ʟ-1-deoxymannojirimycin ((2*S*,3*S*,4*S*,5*S*)-**200**) and ʟ-1-deoxyaltronojirimycin ((2*S*,3*S*,4*R*,5*S*)-**200**). Reagents and conditions: a) MeONHMe, iPrMgCl, THF, 0 °C, 1 h; b) MeC≡CMgBr, THF, −78 °C, 1 h and rt, 1 h; c) NaBH_4_, ZnCl_2_, MeOH, −78 °C, 1 h; d) LiAlH_4_, THF, 0 °C, 0.5 h and reflux, 8 h; e) TBDMSCl, TEA, DMAP, CH_2_Cl_2_, rt, 17 h; f) AcOH, CH_2_Cl_2_, rt, 15 h; g) KOH, EtOH, rt, 0.5 h; h) CDI, DBU, CH_2_Cl_2_, 0 °C, 1 h and rt, 24 h; i) Na, NH_3_ liquid, THF, −78 °C, 0.5 h; j) H_2_CCH=CH_2_I, NaH, ClCH_2_CH_2_Cl, reflux, 10 h; k) Grubbs catalyst (1st generation), CH_2_Cl_2_, rt, 24 h; l) Oxone^®^, NaHCO_3_, Me_2_CO/H_2_O, rt, 0.5 h; m) Me_2_CO, BF_3_·OEt_2_, CH_2_Cl_2_, 0 °C, 2 h; n) LiOH, EtOH/H_2_O, reflux, 4 h; o) HCl, MeOH, reflux, 4 h, then Amberlite IRA-410 OH^¯^; p) OsO_4_, NMO, MeCN/H_2_O, 0 °C to rt, 3 h; r) MCPBA, NaHPO_4_, CH_2_Cl_2_, 0 °C to rt, 72 h.

To synthesize stereoisomeric ʟ-deoxynojirimycins having the *R* configuration at C3 [(2*S*,3*R*,4*R*,5*S*)-**200** (ʟ-1-deoxyidonojirimycin), (2*S*,3*R*,4*R*,5*R*)-**200** (ʟ-1-deoxygulonojirimycin) and (2*S*,3*R*,4*S*,5*R*)-**200** (ʟ-1-deoxygalactonojirimycin)] the aziridine alcohol (2*S*,1'*S*,1''*R*)-**202** prepared by reduction of the ketone (2*S*,1'*R*)-**201** with ʟ-Selectride^®^ was transformed into the bicyclic intermediate **210**, the epimer of **205**, as already shown (Scheme 53) [88]. Furthermore, syntheses of six stereoisomers of ᴅ-1-deoxynojirimycins were accomplished starting from the ester (2*R*,1'*R*)-**5b** and proceeding as already shown in Scheme 53 [88].

**Scheme 54:**
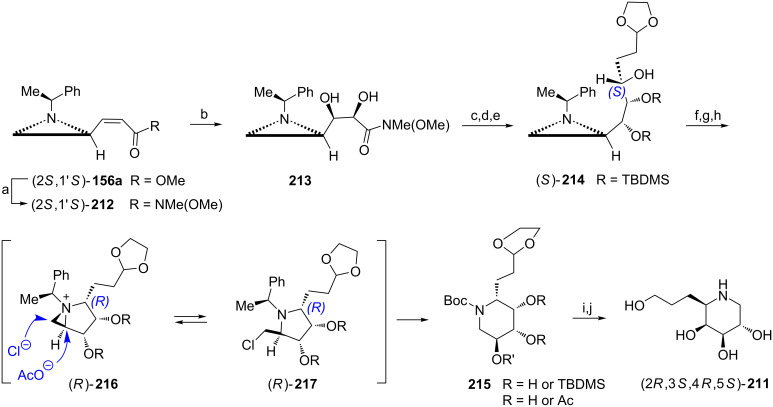
Syntheses of 1-deoxy-ᴅ-*galacto*-homonojirimycin (2*R*,3*S*,4*R*,5*S*)-**211**. Reagents and conditions: a) MeONHMe, iPrMgCl, THF, 0 °C, 15 min; b) OsO_4_, NMO, acetone, 0 °C to rt, 6 h; c) TfOTBDMS, 2,6-lutidine, CH_2_Cl_2_, −78 °C to rt, 2 h; d) DIBAL-H, hexane, −40 °C, 8.5 h; e) 2-(2-bromoethyl)-1,3-dioxolane, Mg, THF, rt, 0.5 h; f) MsCl, TEA, DMAP, CH_2_Cl_2_, 0 °C to reflux, 2 h; g) AcOCs, DMF, 100 °C, 3 h; h) H_2_, 20% Pd/C, Boc_2_O, MeOH, rt, 12 h; i) Et_3_SiH, TFA/CH_2_Cl_2_/H_2_O, rt, 12 h; j) HCl, MeOH, reflux, 2 h then Amberlite 410 Cl.

Enantioselective syntheses of 1-deoxy-ᴅ-*galacto*-homonojirimycin ((2*R*,3*S*,4*R*,5*S*)-**211**) as well as pyrrolizidine alkaloids were completed from the *cis*-acrylate (2*S*,1'*S*)-**156a** (Scheme 54) [34]. Dihydroxylation of Weinreb amide (2*S*,1'*S*)-**212** gave the diol **213** with excellent diastereoselectivity (>99:1) which already had the correct configurations at C3 and C4 of the final product (3*S*,4*R*). After silylation of the hydroxy groups the protected three-carbon moiety was attached by the enantiospecific addition of the Grignard reagent to the respective aldehyde to give the aziridine alcohol (*S*)-**214**. Sequential treatment of (*S*)-**214** with mesyl chloride and cesium acetate followed by catalytic hydrogenation furnished substituted piperidines **215**. Hydrolysis of the dioxolane acetal in **215** in the presence of a reducing agent provided 1-deoxy-ᴅ-*galacto*-homonojirimycin ((2*R*,3*S*,4*R*,5*S*)-**211**). Undoubtedly after mesylation of the alcohol (*S*)-**214** the intermediary bicyclic aziridinium ion (*R*)-**216** was formed which can equilibrate with a 2-chloromethylpyrrolidine (*R*)-**217**. For steric reasons in the presence of cesium acetate ring expansion in (*R*)-**216** took place to produce **215**.

**Scheme 55:**
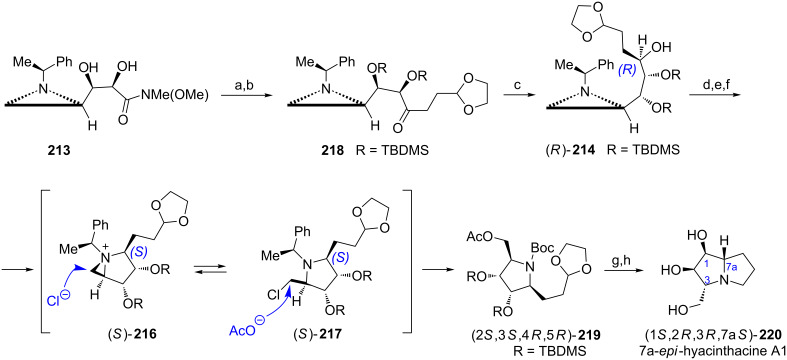
Syntheses of 7a-*epi*-hyacinthacine A1 (1*S*,2*R*,3*R*,7a*S*)-**220**. Reagents and conditions: a) TfOTBDMS, 2,6-lutidine, CH_2_Cl_2_, −78 °C to rt, 2 h; b) 2-(2-bromoethyl)-1,3-dioxolane, Mg, THF, rt, 1 h; c) ʟ-Selectride^®^, CH_2_Cl_2_, −10 °C, 1.5 h; d) MsCl, TEA, DMAP, CH_2_Cl_2_, 0 °C to reflux, 2 h; e) AcOCs, DMF, 100 °C, 3 h; f) H_2_, 20% Pd/C, Boc_2_O, MeOH, rt, 12 h; g) Et_3_SiH, TFA/CH_2_Cl_2_/H_2_O, rt, 12 h; h) HCl, MeOH, reflux, 2 h then Amberlite 410 Cl.

**Pyrrolizidines:** Pyrrolizidine alkaloids [112], e.g., (+)-hyacinthacine A1 [113] were found in many plants and they are primarily recognized as inhibitors of glycosidases [113–114]. To synthesize a hyacinthacine A1 framework the aziridine ketone **218** was stereoselectively reduced to the alcohol (*R*)-**214** which accommodated three stereogenic centers (1*S*,2*R*,3*R*) of the final pyrrolizidine [34]. When (*R*)-**214** was first reacted with mesyl chloride then with cesium acetate and finally was subjected to catalytic hydrogenation the pyrrolidine (2*S*,3*S*,4*R*,5*R*)-**219** was obtained. The formation of the pyrrolizidine skeleton was accomplished as already shown (Scheme 55) to produce (+)-7a-*epi*-hyacinthacine A1 ((1*S*,2*R*,3*R*,7a*S*)-**220**). As for the alcohol (*S*)-**214** (Scheme 54) mesylation of the alcohol (*R*)-**214** gave the bicyclic aziridinium ion (*S*)-**216** which for steric reasons was transformed into a stable chloromethylpyrrolidine (*S*)-**217**. In the presence of cesium acetate the chloride for acetate displacement occurred to yield the pyrrolidine (2*S*,3*S*,4*R*,5*R*)-**219**.

**Scheme 56:**
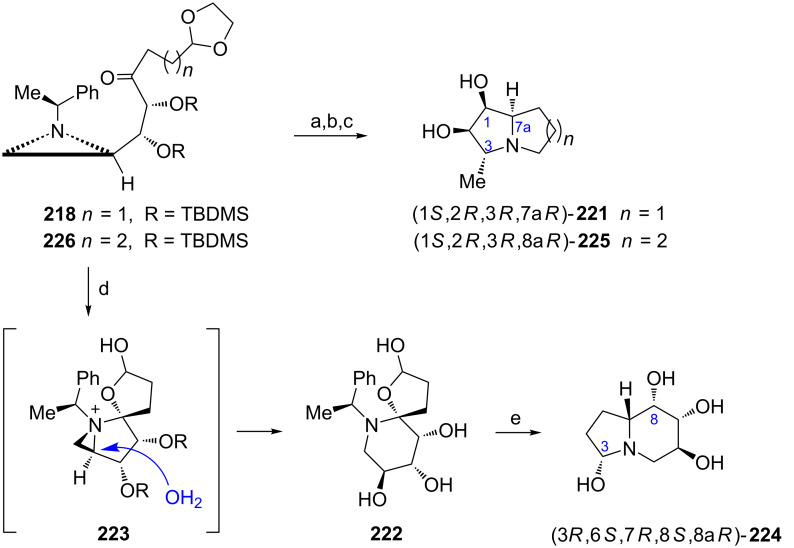
Syntheses of 8-deoxyhyacinthacine A1 ((1*S*,2*R*,3*R*,7a*R*)-**221**). Reagents and conditions: a) H_2_, Pd/C, PTSA, AcOH, rt, 12 h then TFA, rt, 2 h; b) Et_3_SiH, TFA/CH_2_Cl_2_/H_2_O, rt, 12 h; c) HCl, MeOH, reflux, 2 h then Amberlite 410 Cl; d) TFA/H_2_O, rt, 48 h; e) polymethylhydrosiloxane (PMHS), Pd/C, MeOH, reflux, 1 h.

The enantiospecific synthesis of 8-deoxyhyacinthacine A1 ((1*S*,2*R*,3*R*,7a*R*)-**221**) started from the aziridine ketone **218** (Scheme 56) which already had the correct configurations at C1, C2 and C3 of the final product (1*S*,2*R*,3*R*) [34]. Catalytic hydrogenation of **218** in the presence of strong acids combined with treatment with triethylsilane and final acidic hydrolysis gave 8-deoxyhyacinthacine A1 ((1*S*,2*R*,3*R*,7a*R*)-**221**) since even in the presence of acetic acid the Me–C3 group was formed by the reductive opening of the aziridine ring and the first reductive amination appeared to be stereospecific.

**Indolizidines:** Indolizidine alkaloids represent another large group of natural products of manifold biological activities [115]. For example, swainsonine is known as mannosidase inhibitor and it also has a potential in chemotherapy [116]. Castanospermine inhibits several glucosidases and also shows anticancer and antiviral activities [117–118]. (+)-Lentiginosine revealed a potent and selective amyloglucosidase and Hsp90 inhibitory activity [119]. Several synthetic strategies to polyhydroxyindolizidines have been elaborated including procedures employing 2-substituted (1-phenylethyl)aziridines. Thus, in the presence of a strong acid the aziridine ketone **226** was transformed into the piperidine **222** because water attacked C2 in the intermediate bicyclic aziridinium ion **223** from the less hindered side (Scheme 56) [34]. *N*-Debenzylation caused a five-membered ring closure which occurred in a stereospecific manner to form (*R*)-3-hydroxy-1-deoxy-8-*epi*-castanospermine ((3*R*,6*S*,7*R*,8*S*,8a*R*)-**224**).

(3*R*)-3-Methyl-8-deoxyswainsonine ((1*S*,2*R*,3*R*,8a*R*)-**225**) was obtained from the aziridine ketone **226** following strategy applied in synthesis of (1*S*,2*R*,3*R*,7a*R*)-**221** (Scheme 56) [34].

**Scheme 57:**
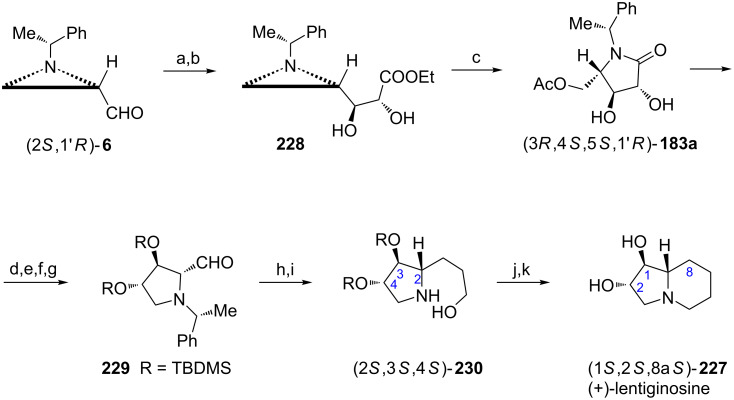
Syntheses of (+)-lentiginosine ((1*S*,2*S*,8a*S*)-**227**). Reagents and conditions: a) (EtO)_2_P(O)CH_2_COOEt, LiHMDS, THF, rt, 1 h; b) AD-mix-α, MeSO_2_NH_2_, *t*-BuOH/H_2_O, 0 °C, 24 h; c) AcOH, CH_2_Cl_2_, rt, 12 h, then toluene, 90 °C, 12 h; d) TfOTBDMS, 2,6-lutidine, CH_2_Cl_2_, 0 °C, 1 h; e) KOH, EtOH, rt, 1 h; f) BH_3_·SMe_2_, THF, 0 °C to 70 °C, 3 h; g) Swern oxidation; h) Ph_3_P^+^(CH_2_)_3_OBn Brˉ, *t*-BuOK, THF, 0 °C to rt, 1 h; i) H_2_, Pd(OH)_2_, CF_3_COOH, MeOH, rt, 24 h; j) CBr_4_, Ph_3_P, TEA, CH_2_Cl_2_, 0 °C to rt, 6 h; k) HCl, MeCN, rt, 4 h.

To construct a five-membered ring of (+)-lentiginosine ((1*S*,2*S*,8a*S*)-**227**) the aziridine aldehyde (2*S*,1'*R*)-**6** was subjected to HEW olefination followed by asymmetric Sharpless dihydroxylation to give the major (11:1) ester **228** which was transformed into the pyrrolidin-2-one (3*R*,4*S*,5*S*,1'*R*)-**183a** in two standard steps (Scheme 57) [32]. Before installation of a three-carbon moiety hydroxy groups in (3*R*,4*S*,5*S*,1'*R*)-**183a** were protected, the acetate hydrolyzed, an amide carbonyl reduced and the hydroxymethyl residue oxidized to provide the pyrrolidine aldehyde **229**. After Wittig reaction and hydrogenation, the 4-hydroxybutyl chain was installed in the protected pyrrolidine (2*S*,3*S*,4*S*)-**230**. The piperidine ring closure was carried out with an Appel reaction while treatment with acids afforded (1*S*,2*S*,8a*S*)-octahydroindolizine-1,2-diol (1*S*,2*S*,8a*S*)-**227**.

**Scheme 58:**
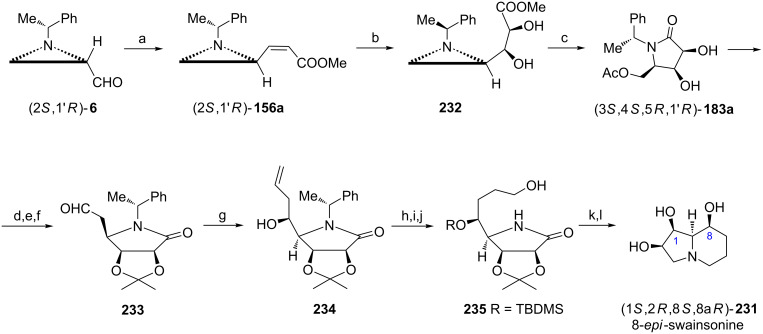
Syntheses of 8-*epi*-swainsonine (1*S*,2*R*,8*S*,8a*R*)-**231**. Reagents and conditions: a) Ph_3_P=CHCOOMe, MeOH, 0 °C, 1 h; b) AD-mix-β, MeSO_2_NH_2_, *t*-BuOH/H_2_O, rt, 24 h; c) AcOH, CH_2_Cl_2_, rt, 12 h, then toluene, 90 °C, 12 h; d) Me_2_C(OMe)_2_, PTSA, CH_2_Cl_2_, rt, 1 h; e) KOH, MeOH, rt, 1 h; f) Dess–Martin periodinane, CH_2_Cl_2_, rt, 2 h; g) H_2_C=CHCH_2_MgBr, (−)-Ipc_2_BOMe, CH_2_Cl_2_/ether, −78 °C to 0 °C, 3 h; h) TfOTBDMS, 2,6-lutidine, CH_2_Cl_2_, 0 °C to rt, 2 h; i) BH_3_·SMe_2_, THF, 0 °C to rt, 3 h then H_2_O_2_, NaOH, MeOH, 0 °C; j) H_2_, Pd(OH)_2_, MeOH, rt, 6 h; k) CBr_4_, Ph_3_P, CH_2_Cl_2_, NEt_3_, rt, 3 h; l) TFA, CH_2_Cl_2_, 40 °C, 4 h.

The elegant approach to 8-*epi*-swainsonine ((1*S*,2*R*,8*S*,8a*R*)-**231**) started from *cis*-acrylate (2*S*,1'*R*)-**156a** readily separable from a 4:1 *cis*:*trans* mixture prepared in Wittig reaction from the aldehyde (2*R*,1'*R*)-**6** (Scheme 58) [120]. Sharpless asymmetric dihydroxylation of (2*S*,1'*R*)-**156a** provided a 10:1 mixture of diols with the diastereoisomer **232** predominating. Opening of the aziridine ring with acetic acid caused cyclization to the substituted pyrrolidin-2-one (3*S*,4*S*,5*R*,1'*R*)-**183a**. Protection of the diol moiety, ester hydrolysis and Dess–Martin oxidation afforded the aldehyde **233** which was used as a starting material in asymmetric Brown allylation to form a 4:1 mixture of diastereoisomers and the major one **234** was separated chromatographically. Silylation preceded treatment with borane to execute reduction of the carbonyl group and to form a primary alcohol and was followed by the removal of the chiral auxiliary to give **235**. A six-membered ring closure in the alcohol **235** was carried out by Appel reaction to produce 8-*epi*-swainsonine ((1*S*,2*R*,8*S*,8a*R*)-**231**) after acidic deprotection.

**Scheme 59:**
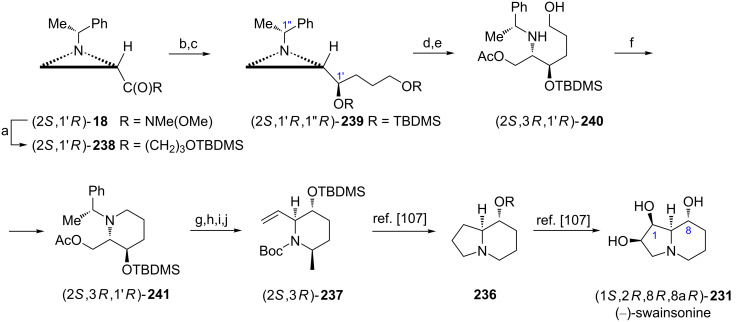
Synthesis of a protected vinylpiperidine (2*S*,3*R*)-**237**, a key intermediate in the synthesis of (−)-swainsonine ((1*S*,2*R*,8*R*,8a*R*)-**231**). Reagents and conditions: a) Mg, Br(CH_2_)_3_OTBDMS, THF, reflux, 8 h; b) NaBH_4_, ZnCl_2_, MeOH, −78 °C, 1.5 h; c) TBDMSCl, DMAP, CH_2_Cl_2_, 0 °C to rt, 12 h; d) AcOH, CH_2_Cl_2_, rt, overnight; e) AcOH, THF/H_2_O, rt, 48 h; f) MsCl, TEA, CH_2_Cl_2_, 0 °C to rt, 24 h; g) H_2_, Pd(OH)_2_, Boc_2_O, MeOH, 100 psi, rt, 5 h; h) KOH, MeOH, rt, 0.5 h; i) Swern oxidation; j) Ph_3_PMe^+^Br¯, LiHMDS, THF, 0 °C to rt, 1 h.

Several syntheses of (−)-swainsonine ((1*S*,2*R*,8*R*,8a*R*)-**231**) have been described including an approach relying on the *cis*-dihydroxylation of the intermediate **236** which was prepared from the vinylpiperidine (2*S*,3*R*)-**237** (Scheme 59) [121]. Synthesis of (2*S*,3*R*)-**237** started with a stereoselective reduction (>99:1) of the aziridine ketone (2*S*,1'*R*)-**238** to give a protected aziridine diol (2*S*,1'*R*,1''*R*)-**239** [122]. After the aziridine ring opening with acetic acid and selective desilylation to form (2*S*,3*R*,1'*R*)-**240** the piperidine ring closure was achieved by mesylation producing (2*S*,3*R*,1'*R*)-**241**. The final steps to the key intermediate (2*S*,3*R*)-**237** included removal of the chiral auxiliary, deacetylation, Boc protection, Swern oxidation of the hydroxymethyl group and methylenation with Wittig reagent.

#### Miscellaneous

**Scheme 60:**
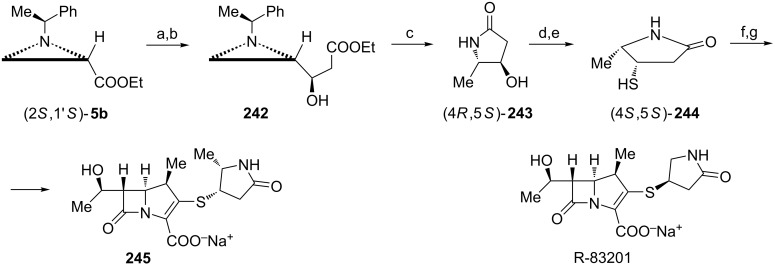
Synthesis of a modified carbapenem **245**. Reagents and conditions: a) AcOEt, LiHMDS, THF, −78 °C, 1.5 h; b) NaBH_4_, NH_4_Cl, EtOH/H_2_O, rt, 1 h; c) H_2_, Pd(OH)_2_, EtOH, rt, 4.1 bar, 12 h; d) AcSH, Ph_3_P, DEAD, THF/DMF, rt, 2 h; e) NaOH, MeOH, 0 °C, 3 min; f) 4-nitrobenzyl (1*R*,5*S*,6*S*)-2-diphenylphosphoryloxy-6-[(*R*)-1-hydroxyethyl]-1-methyl-1-carbapen-2-em-3-carboxylate, DIPEA, MeCN, 0–5 °C, 2 h; g) H_2_, Pd/C, THF/EtOH/Mops buffer, rt, 15 h.

FDA-approved meropenem and R-82301 belong to a class of carbapenem antibiotics containing a substituted exocyclic 3-mercaptopyrrolidine scaffold. In search for new drugs this fragment was replaced by a 5-methyl-4-mercaptopyrrolidin-2-one moiety which was synthesized from aziridine esters **5** [123]. Thus, a five-carbon framework was assembled from (2*S*,1′*S*)-**5b** and ethyl acetate followed by reduction of an intermediary ketone which produced a diastereoisomeric pair of aziridine alcohols readily separable chromatographically. Hydrogenation of, e.g., the alcohol **242** led to the formation of the pyrrolidine-2-one (4*R*,5*S*)-**243** (Scheme 60). The hydroxy for mercapto replacement was achieved in Mitsunobu reaction to produce (4*S*,5*S*)-**244** after deacetylation. Three other enantiomers were prepared accordingly. Modified carbapenems, e.g., **245**, were obtained by coupling of the thiol (4*S*,5*S*)-**244** with the respective enolphosphate and some of them appeared as active as meropenem and R-82301 towards selected bacterial strains.

## Conclusion

The current body of literature on *N*-(1-phenylethyl)aziridine-2-carboxylate esters and their derivatives revealed their successful applications in syntheses of over a dozen of approved drugs and potential medications as well as over 40 important natural products mostly alkaloids including ephedrine and related compounds, polyhydroxy pyrrolidines, piperidines, pyrrolizidines and indolizidines but also various sphingoids and ceramides and their 1- and 3-deoxy analogues and several hydroxy amino acids and their precursors. Designed strategies provided new procedures to several drugs and alternative approaches to natural products. This was possible because three-carbon aziridine chirons **5**–**7** are configurationally stable, in majority of cases the openings of the aziridine ring took place at the less substituted C3 atom, either alkoxycarbonyl or hydroxymethyl but especially formyl functionalities could be further elaborated in a highly stereoselective manner while the cleavage of the chiral auxiliary could be easily accomplished with three different reagents depending on a particular stage of multi-step sequences.

Since a vicinal amino alcohol unit and various amino(dihydroxy)propyl and (diamino)hydroxypropyl combinations are crucial for biologically active compounds (2*R*)- or (2*S*)-aziridine-2-carboxylates, -carbaldehydes and -methanols appeared perfectly suited for introducing the required stereochemistry into a C*–N moiety of the target compound without fear of racemization. The next stereogenic center was best created by reductions of 2-acyl-*N*-(1-phenylethyl)aziridines which proceeded in almost diastereospecific manner when a NaBH_4_/ZnCl_2_ mixture or ʟ-Selectride^®^ were used to give alcohols of opposite configurations. To install at C2 the 1,2-dihydroxyalkyl substituent of required configurations the *cis*-dihydroxylation of *E*- or *Z*-olefins obtained in Wittig or HWE reactions appeared a method of choice. Although Michael reaction on the respective *E*- or *Z*-acylates could introduce various nucleophiles (C, N, O), the stereoselectivity of additions in most cases was not satisfactory. Summing up, a majority of strategies relied on specific functionalization at the C2 site of starting aziridines.

Expansions of a three-carbon framework of aziridines **5**–**8** were also carried out starting from regioselective openings at less substituted C3 with appropriate nucleophiles combined with further functionalizations but were less common and no stereogenic center was generated. Furthermore, hydrogenolytic cleavage of the aziridine ring always occurred at C3 and allowed for the one-step introduction of the terminal methyl group. Amines also reacted preferentially at C3 leading to formation of 1,2-diamino- or 1,2-diamino-3-propyl fragments of designed stereochemistry extremely useful in the synthesis of new sphingoid and ceramide analogues for biological studies. Although hydroxylations also took place at C3, they were carried out as a two-step process (acetylation and saponification).

Openings of the aziridine ring at C2 in 2-substituted *N*-(1-phenylethyl)aziridines were observed in a few cases and were limited to a Lewis acid-catalyzed five-membered ring closure and the aziridine ring opening sequence as well as to reactions at C2–COOEt.

Regardless of regioselectivity the aziridine ring opening in *N*-(1-phenylethyl)aziridines required prior activation to form respective aziridinium ions. Besides protic (AcOH, HF) and Lewis acids (aluminum chloride, boron trifluoride etherate), chlorotrimethylsilane–amine pairs, iodotrimethylsilane, phosgene and triphosgene were applied to mention frequently used. However, when bicyclic aziridinium ions are formed their further reactivity appeared to be under steric and/or electronic control and followed rules recently described [124–125].

On a few occasions only epimers of naturally occurring compounds were obtained without any comments on modification of the synthetic strategy to reach the target molecule. With a collection of reliable transformations of 2-substituted *N*-(1-phenylethyl)aziridines in hands new applications in enantioselective syntheses of medications and natural products will appear soon and we hope this review will stimulate further research in this area.
